# Pharmacological inhibition of RAS overcomes FLT3 inhibitor resistance in FLT3-ITD+ AML through AP-1 and RUNX1

**DOI:** 10.1016/j.isci.2024.109576

**Published:** 2024-03-26

**Authors:** Daniel J.L. Coleman, Peter Keane, Paulynn S. Chin, Luke Ames, Sophie Kellaway, Helen Blair, Naeem Khan, James Griffin, Elizabeth Holmes, Alexander Maytum, Sandeep Potluri, Lara Strate, Kinga Koscielniak, Manoj Raghavan, John Bushweller, Olaf Heidenreich, Terry Rabbitts, Peter N. Cockerill, Constanze Bonifer

**Affiliations:** 1 Institute of Cancer and Genomic Sciences, University of Birmingham, Birmingham, UK; 2 Wolfson Childhood Cancer Research Centre, University of Newcastle, Newcastle upon Tyne, UK; 3 Institute of Immunology and Immunotherapy, University of Birmingham, Birmingham, UK; 4 School of Medicine, University of Virginia, Charlottesville, VA, USA; 5 Princess Máxima Centrum of Pediatric Oncology, Utrecht, the Netherlands; 6 Division of Cancer Therapeutics, The Institute of Cancer Research, London, UK

**Keywords:** Pharmacy, Molecular biology

## Abstract

AML is characterized by mutations in genes associated with growth regulation such as internal tandem duplications (ITD) in the receptor kinase FLT3. Inhibitors targeting FLT3 (FLT3i) are being used to treat patients with FLT3-ITD+ but most relapse and become resistant. To elucidate the resistance mechanism, we compared the gene regulatory networks (GRNs) of leukemic cells from patients before and after relapse, which revealed that the GRNs of drug-responsive patients were altered by rewiring their AP-1-RUNX1 axis. Moreover, FLT3i induces the upregulation of signaling genes, and we show that multiple cytokines, including interleukin-3 (IL-3), can overcome FLT3 inhibition and send cells back into cycle. FLT3i leads to loss of AP-1 and RUNX1 chromatin binding, which is counteracted by IL-3. However, cytokine-mediated drug resistance can be overcome by a pan-RAS inhibitor. We show that cytokines instruct AML growth via the transcriptional regulators AP-1 and RUNX1 and that pan-RAS drugs bypass this barrier.

## Highlights

• FLT3 inhibition in patients with AML blocks AP-1 and RUNX1 chromatin binding
• Relapse after FLT3 inhibition is caused by rewiring of MAP Kinase signaling
• Rewiring is caused by RAS-dependent alternate growth factor action
• RAS and RUNX1 inhibition block MAP Kinase rewiring

## Graphical abstract

**Figure:**
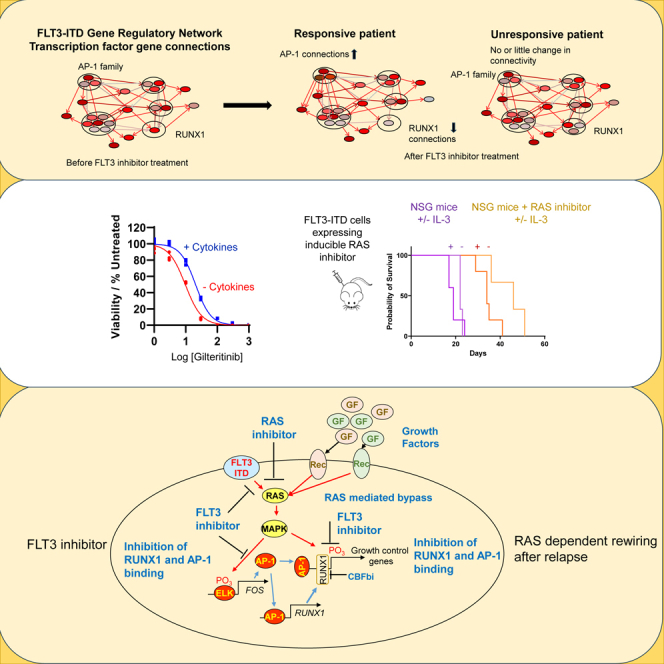

## Introduction

Acute myeloid leukemia (AML) occurs when mutations in immature myeloid stem or progenitor cells lead to a block in differentiation and an increase in proliferation.^1^^,^^2^ They can broadly be categorized as mutations in transcription factors (TFs), epigenetic regulators, and signaling genes. FMS (Feline-McDonough-Sarcoma)-like tyrosine kinase 3 (FLT3) is one of the most commonly mutated genes in AML. It is detected in 30% of cases, with the most common type of mutation being the internal tandem duplication (ITD), which occurs in 25% of AMLs^3^^,^^4^ This mutation confers a particularly poor prognosis, with less than 60% of patients reaching complete remission and an overall relapse rate of 77%.^5^ Due to the aggressive nature of this AML sub-type, inhibitors which directly target FLT3, such as Gilteritinib^6^ have been developed and approved for use in a clinical setting. However, while Gilteritinib improves the overall survival of patients in comparison to salvage chemotherapy (median survival of 9.3 and 5.6 months, respectively), the relapse rates of patients taking Gilteritinib is 76%.^7^

One mechanism by which AML cells can become resistant to FLT3 inhibition is through the development of mutations, either those occurring in FLT3 directly such as the F691L mutation which prevents the binding of Gilteritinib to the FLT3 protein,^8^ or newly developed activating mutations in downstream targets of FLT3, including MAPK pathway genes such as *NRAS or KRAS,* which have been detected in 38.9% of relapse patients.^9^ FLT3-ITD signals specifically through KRAS to activate MAPK signaling whereas WT FLT3 signals through both NRAS and KRAS,^10^ therefore activating mutations in these genes will restore the constitutive MAPK signaling lost with the inhibition of FLT3. However, not all samples from patients who relapse after FLT3 inhibition have detectable genetic mutations^9^ suggesting that alternative mechanisms could bypass the lost signaling of inhibited FLT3-ITD.^11^^,^^12^ Such mechanisms of resistance would be especially difficult to target by conventional treatments. Any treatment specifically targeting alternate surface receptors would still be vulnerable to the AML rewiring its signaling. What is required in this situation is the inhibition of shared central signaling molecules or TFs downstream of all relevant growth factor receptor pathways.

To address this issue, we constructed gene regulatory networks (GRNs) to highlight which TFs regulate which genes. GRNs were constructed from patient samples taken before and after treatment with FLT3 inhibitors (FLT3i) and subsequent relapse. AP-1, which is a heterodimer between JUN and FOS protein families, is a main mediator of MAP-Kinase signaling by binding to chromatin in a signaling-responsive fashion.^13^ RUNX1 is required for hematopoietic stem cell development^13^ and regulates a multitude of genes required for blood cell development and growth. GRN comparison showed that the binding of AP-1 and RUNX1 changes substantially after FLT3i relapse resulting in the upregulation of genes associated with numerous different signaling pathways. Activation of these signaling pathways with multiple cytokines suppressed the effects of FLT3i on gene regulation and cellular viability. For one cytokine, IL-3, we demonstrate that it counteracts FLT3i-driven loss of RUNX1 chromatin binding and restores leukemic growth. To bypass this mechanism of resistance to FLT3 inhibitors, we employed a pan-RAS inhibitor that blocks cytokine-mediated signaling rewiring and restores RUNX1 binding, thus efficiently targeting leukemic growth both *in vitro* and *in vivo*.

## Results

### AP-1 and RUNX1 transcription factors rewire the FMS-like tyrosine kinase 3-internal tandem duplications gene regulatory networks in response to FMS-like tyrosine kinase 3 inhibitors

We hypothesized that AML GRNs would rewire following relapse from FLT3i treatment, and that knowledge of these GRNs would reveal molecular mechanisms underlying drug resistance. We therefore performed DNaseI-seq on CD34^+^ AML cell chromatin from patients with FLT3-ITD and identified the occupied TF binding motifs as described in.^1^^,^^14^ Using FLT3-ITD-specific promoter capture HiC data from a reference FLT3-ITD+ AML sample from the same study, we constructed GRNs from purified leukemic blast cells of FLT3-ITD+ AML samples from patients before treatment and after relapse from treatment with FLT3i. We also generated RNA-Seq data from such cells from all three patients. All three patients maintained the FLT3-ITD mutation at relapse (Figure S1A). One patient (ITD15) was treated with the FLT3i Quizartinib,^15^ while the others (ITD16 and ITD17) were treated with Gilteritinib.^6^ Patient ITD15 relapsed after FLT3i with a D835H tyrosine kinase domain mutation, which blocks the binding of Quizartinib,^16^ whereas ITD16 and ITD17 had no additional detectable mutational changes after relapse. Importantly, in contrast to the two other patients, ITD17 showed no significant response to drug treatment, with relapse occurring after only 3 months of treatment.

**Figure 1:**
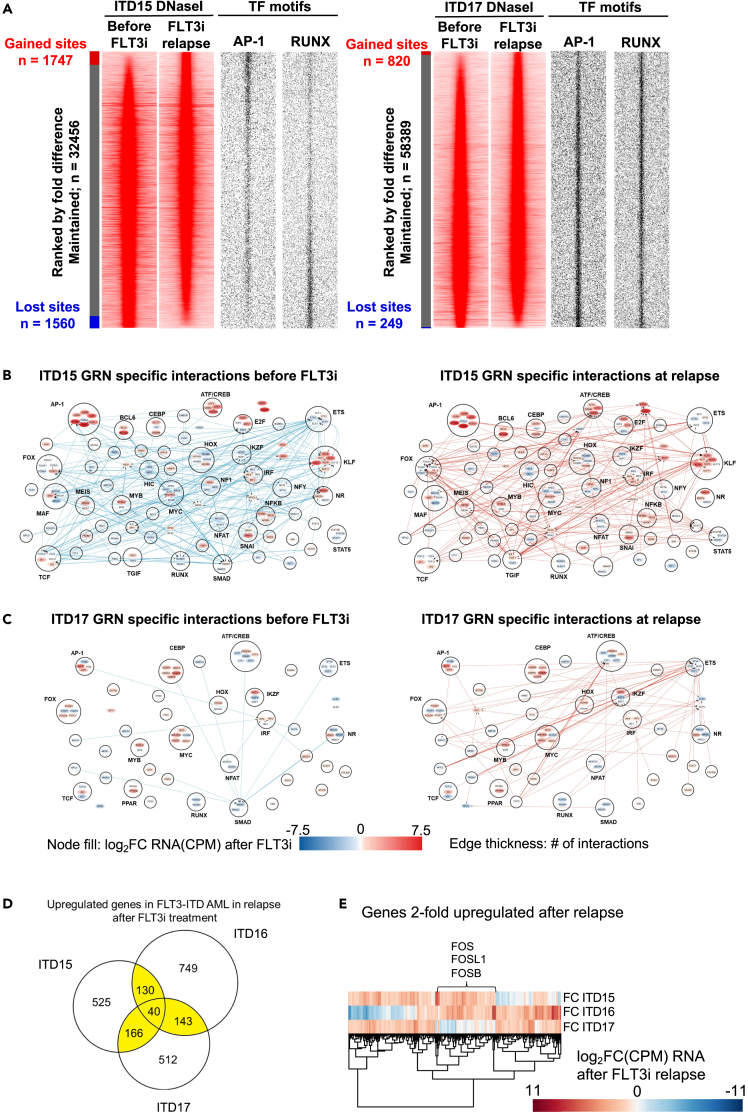
Relapse samples from patients treated with FLT3i show rewiring of the gene regulatory network (A) Left panels: DNaseI density plots for patients treated with FLT3i ITD15 (responder) shows more sites changing than ITD17 (non-responder). Open chromatin regions changing more that 3-fold are shown. Right panels: TF binding motifs enriched in DNaseI hypersensitive sites. (B and C) TF networks of ITD15 (B) and ITD18 (C). Specific interactions before FLT3i (blue edges left) and after relapse from FLT3i (red edges right) represent DHSs changing 3-fold in either sample. Nodes represent TF families binding to the same sequence, their fold change of RNA expression in relapse after FLT3i compared to the diagnostic sample indicated in red, with TFs in families with connections included if they change >2-fold. (D) Venn diagram showing the Fold change of RNA expression of the upregulated gene overlap after FLT3i treatment relapse in two responsive (ITD15 and ITD16) and 1 non-responsive patient (ITD17). Genes were filtered for the AML specificity of expression (>2-fold UP vs. PBSC) prior to analysis. Genes upregulated in >1 sample (highlighted in yellow) were used for further analysis. (E) Heatmap showing unsupervised clustering of highlighted genes in (D). Genes upregulated in the responsive patients and unchanged in the non-responsive patient are highlighted. These include several AP-1 family members.

The DNaseI-Seq analyses revealed substantial changes in the chromatin accessibility pattern of ITD15 after relapse, but few changes in the non-responsive patient ITD17 (Figure 1A, no data available for ITD16). DNaseI hypersensitive sites (DHSs) specific for the ITD15 FLT3i relapse sample (1747 sites 3-fold changed) were enriched in AP-1 motifs, whereas RUNX motifs were enriched in depleted DHSs (1560 sites 3-fold changed). Only 820 and 249 sites changed 3-fold up or down, respectively in ITD17. However, despite the differences in the regions of open chromatin, all 3 patients showed similar numbers of genes changing expression (>2-fold change) between presentation and relapse (Figure S1B), suggesting that the differences in drug responsiveness of the two patients were encoded in the gene regulatory phenotype.

To understand the differences between ITD15 and ITD17 at the gene regulation level, we compared the GRNs for all four samples against each other to see what had changed.^1^ Figures 1B and 1C show the regulatory interactions between transcription factors (TFs) and their target genes, with the edges between nodes indicating the presence of open regions of chromatin with the TF motif from the source TF family node present on the target gene node. The fold change in the gene expression of the individual genes after FLT3i relapse is indicated by the node color. Genes were included in the network as nodes when there were direct connections to the gene or when the gene was >2-fold deregulated after FLT3i relapse, and the TF was included in a TF family source node. The networks show interactions specific for presentation (blue connections) and after relapse (red connections). Note that for visualization purposes, we only show connections between genes encoding for TFs, the entire network is much larger than depicted (Data S1).

The GRN of patient ITD15 shows extensive rewiring after relapse from FLT3i treatment, with numerous changes in connections between TFs and TF encoding genes and their downstream non-TF targets. The AP-1 TF family becomes a key node in the relapse-specific network, with 8 connections to other TF genes specific to the relapse network, whilst 4 connections are lost at relapse. When AP-1 connections to all genes were considered, rewiring was even more prominent (Figure S1C, Data S1 (TF network matrices)), with 314 connections gained at relapse, and 76 connections lost. In contrast, in ITD17 after relapse, hardly any rewiring occurs, for the AP-1 node only 2 connections with other TFs were gained after relapse. For all genes, 66 additional connections were gained, while 22 interactions were lost. These observations indicate that patient ITD17 may be unresponsive to FLT3i treatment due to an already increased expression of AP-1 family genes prior to treatment. To confirm this result we overlapped the gene expression changes in the three FLT3i-treated patient samples at relapse after filtering for AML-specific genes by selecting genes >2-fold upregulated in at least one of the patient samples compared to a previously published RNA-Seq data from CD34^+^ cells from peripheral blood^1^ (Figure 1D). By comparing the gene expression fold-changes (FC) at relapse of genes upregulated in at least 2 of our patients by unsupervised clustering, we identified a group of genes which were upregulated in the FLT3i responsive patients (ITD15 and ITD16) but not in ITD17 (Figures 1E and S1D). This group of genes included three members of the AP-1 family, which were upregulated at relapse in the FLT3i responsive patients but were already highly expressed in ITD17 (Figure S1E). The pattern of down-regulated genes in the two responding patients partially overlapped but was more heterogeneous (Figure S1F).

**Figure 2:**
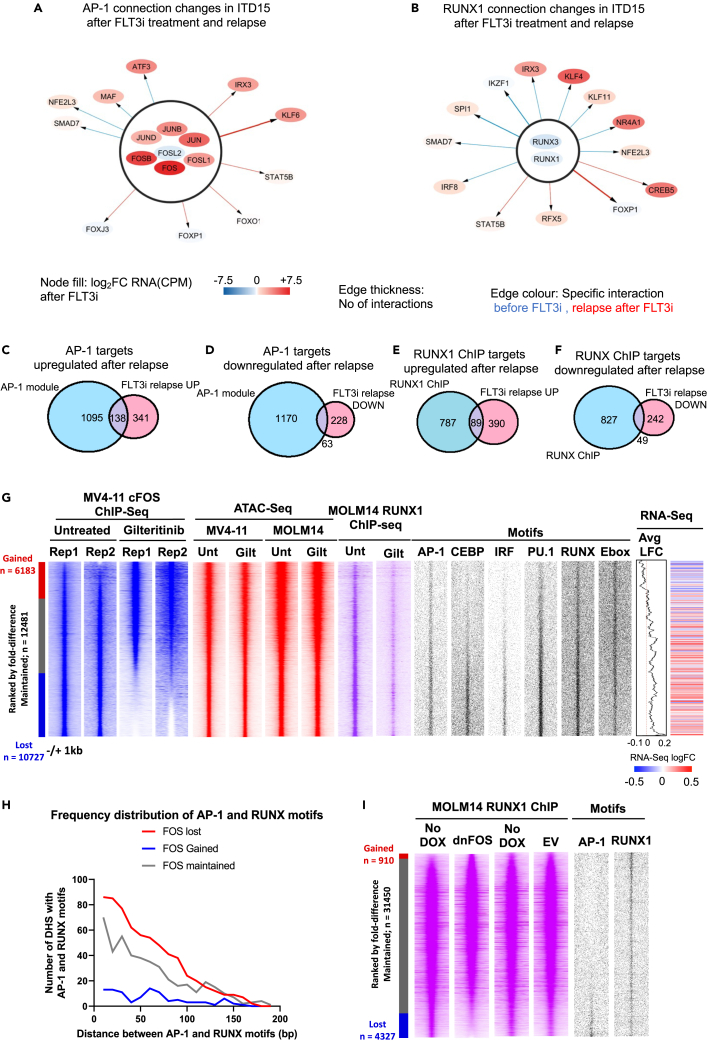
FLT3 inhibition reduces AP-1 and RUNX1 binding leading to deregulation of target genes (A andB) Changing connections within the AP-1 (A) RUNX1 (B) module after FLT3i relapse of ITD15. Blue edges show connections lost at relapse, red edges show gained connections. Node color indicates the fold change of RNA expression at relapse as compared to the diagnostic sample. (C and D) Venn diagram showing overlap of AP-1 family target genes (by motifs in sites connected by HiC) and genes upregulated (C) or downregulated (D) in >1 FLT3i relapse samples. (E and F) Venn diagram showing overlap of RUNX1 ChIP target genes (in sites connected by HiC) and genes upregulated (E) or downregulated (F) in >1 FLT3i relapse samples. (G) Genome wide analysis of FOS ChIP in MV4-11 cells after treatment with 10 nM Gilteritinib. Density plots are ranked by the fold-change of the FOS peak tag count in untreated samples vs. FLT3i (blue). RUNX1 ChIP peaks from MOLM14 are aligned to the same sites (pink) and sites are filtered for ATAC peaks (red). Enriched motif distribution in open chromatin regions is shown in black. The far right panel shows the average logFoldChange (LFC) of the expression of the genes assigned to the peaks in MV4-11 cells treated with FLT3i. (H) Spatial distribution of AP-1 and RUNX motifs in FOS ChIP peaks. (I) Genome wide analysis of RUNX1 ChIP in MOLM14 cells with and without induction of dnFOS. Pink density plots show RUNX1 ChIP ranked by fold change of RUNX1 peak tag count in samples after dox induction, the first two panels show ChIP from pCW57.1-dnFOS MOLM14 with and without dox, with the third and 4^th^ panels showing the pCW57.1-EV controls. AP1 and RUNX1 motif distribution is shown in the black panels.

We next examined the changes in the connections between AP-1, RUNX1 and their targets in more detail (Figures 2A and 2B). To this end, we created an AML-specific GRN of all genes using DHSs which were upregulated >3-fold or maintained in the relapsed FLT3i samples compared to healthy PBSCs1 (Data S1 (TF network matrices)). These analyses revealed that 29% (138) of upregulated genes were part of the AP-1 interaction module, while 22% (63) of genes downregulated at relapse were AP-1 targets (Figures 2C and 2D). KEGG pathway analysis of this gene set showed the upregulation of genes associated with numerous signaling pathways after relapse, including signaling responsive TFs (Figure S2A, Data S2). RUNX loses 12 and gains 7 connections with TF genes after FLT3i relapse including those with *IRX3, CREB5,* and *KLF4* all of which were upregulated at the RNA level (Figure 2B) and globally, RUNX interactions with genes are lost after relapse from FLT3i in ITD15 (Figure S1C). To determine the role of RUNX1 in deregulating genes in FLT3i relapse we overlapped our 2-fold deregulated set of genes after FLT3i relapse with a RUNX1 chromatin-immunoprecipitation (ChIP-Seq) dataset from 2 primary FLT3-ITD+ AML samples and MOLM1414. In this dataset, a gene is judged to be a RUNX1 target if it is bound by RUNX1 in more than one of the samples. This overlap showed that 19% of upregulated genes and 17% of downregulated genes were RUNX1 targets (Figures 2E and 2F). Taken together, our data show that despite a strong response of all patients with regards to gene expression, relapse from FLT3i is associated with a rewiring of the AP-1 and RUNX1 regulatory modules in the drug-responsive patient, but not in the unresponsive patient, suggesting an important role of these TFs in mediating drug response.

### AP-1 and RUNX1 chromatin binding is suppressed by FLT3 inhibition

To confirm whether FLT3 inhibition has a direct effect on AP-1 and RUNX1 binding in chromatin, we performed FOS and RUNX1 ChIP-Seq experiments in FLT3-ITD+ AML cell lines before and after treatment with Gilteritinib (Figure 2G). These data show that after FLT3i treatment AP-1 (FOS) binding was lost from many sites that exhibited enrichment for AP-1, C/EBP, IRF, PU.1, and RUNX motifs (motif panels). Furthermore, the same was true for RUNX1, where extensive loss of binding was seen. The ATAC seq profile of MOLM14 and MV4-11 shows a high degree of similarity when ranked by the fold change in the MV4-11 FOS ChIP, indicating the regions of open chromatin where FOS binding is lost after Gilteritinib treatment are present in both cell lines. Moreover, the enriched motif signature in both AP-1 and RUNX1 bound accessible sites suggested a loss of *cis*-elements binding C/EBP, IRF, and PU.1 factors, which are normally specific for more mature myeloid cells.

We next correlated AP-1 and RUNX1 binding with gene expression changes. The treatment of FLT3-ITD cell lines with FLT3i led to large changes in gene expression (Figures S2B and S2C) with a substantial overlap between MOLM14 and MV4-11 in the subsets of up- and down-regulated genes (Figures S2D and S2E). As seen after relapse (Figure S2A), numerous signaling-associated genes were up-regulated by FLT3i (Figure S2F, left panel), whereas cell cycle and replication genes were down-regulated (Figure S2F, right panel). 850 genes associated with AP-1-bound sites showed increased mRNA levels after FLT3i treatment (Figure S2G), whilst 862 AP-1-bound genes were down-regulated (Figure S2H). Of the genes that lost FOS binding after FLT3i treatment, 32% were upregulated and 21% were down regulated (Figure S2J; Data S2). A parallel analysis of genes bound by RUNX1 prior to FLT3i treatment showed that 22% of these genes were upregulated genes and 12% were downregulated (Figures S2K and S2L). When AP-1 and RUNX1 binding data were correlated together, the sites from which FOS was lost after FLT3 inhibition were more enriched for RUNX1 binding prior to treatment in MOLM14 cells than the sites where FOS binding was not lost (Figures 2G and S2M).

**Figure 3:**
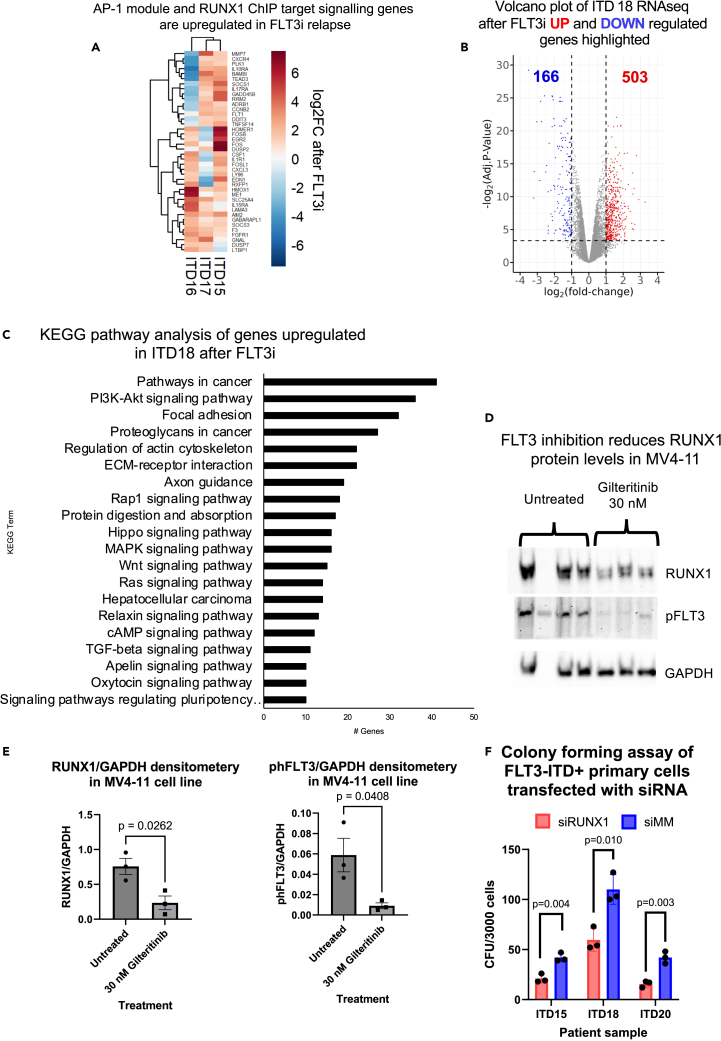
FLT3i operates via RUNX1 and AP-1 regulatory modules (A) Heatmap highlighting signaling genes (determined by KEGG pathway analysis) in >1 patient sample which are members of the AP-1 and/or RUNX1 ChIP module showing log2FC RNA change in expression. (B) Volcano plot of the expression of genes deregulated by Gilteritinib treatment in primary FLT3-ITD AML ITD18. (C) KEGG pathway analysis of genes 2-fold upregulated in ITD18 after Gilteritinib treatment. (D) Western Blot showing RUNX1 destabilisation after 30 nM Gilteritinib treatment. (E) Densitometry analysis of the RUNX1 and phFLT3 signals from Western Blots (D) (n = 3), p values calculated using Student's t test, error bars show standard deviation. (F) Histogram showing colony a formation assay of 3 FLT3-ITD+ primary cell samples transfected with siRNA targeting RUNX1 (red) or mismatch control (blue) (n = 3), p values were calculated using Student’s t test, error bars show standard deviation.

An analysis of the spacing between RUNX and AP-1 motifs revealed that these sites were closer at sites bound prior to FLT3i (Figure 2H) suggesting that the two factors cooperate, and that cooperation is lost after FLT3i treatment. To directly show cooperativity between the two factors, we expressed a Doxycycline-inducible dominant negative, broad specificity FOS peptide^17^ in MOLM-14 cells and performed a RUNX1 ChIP with and without induction (Figure 2I). Induction led to the loss of more than 4000 RUNX1 binding sites, most of which were located near AP-1 motifs. Genes co-bound by AP-1 and RUNX1, and up-regulated after relapse in FLT3i-treated patients (ITD15,16,17) coded for multiple signaling genes (Figures S2N and S2O) and included several cytokine receptor molecules such as IL1R and cytokines such as CSF1, AP-1, and the early growth factor response (EGR) genes (Figures 3A and S1D). The increased protein expression of cytokine receptors could be detected by flow cytometry (Figure S3A).

To further validate the cell line results, we treated cultured primary cells from an additional FLT3-ITD AML patient (ITD18) with Gilteritinib followed by RNA-Seq (Figure 3B). As seen in the cell lines, multiple signaling-associated genes showed increased mRNA levels (Figure 3C). The correlation of AP-1 and RUNX1 ChIP-Seq data with RNA-Seq data from ITD18 confirmed a similar pattern of drug responsiveness as that seen in FLT3-ITD cell lines (Figure S3B). These sites were linked to 98 genes deregulated in both MOLM14 and MV4-11 cells after FLT3 inhibition, which included members of multiple signaling pathways and cell cycle genes (Figures S2O, S2P, S3B, and S3C). These genes include *KIT*, which was also upregulated on the surface of MOLM14 cells treated with Gilteritinib as observed by flow cytometry (Figure S3D).

In summary, our work highlights a strong association of RUNX1 and AP-1 binding with the responsiveness to FLT3i. Moreover, our data show a direct cooperation between the two factors in the genome with multiple overlapping binding sites and a joint loss of binding after FLT3i.

### IL-3 and other cytokines overcome FLT3 inhibition and restore RUNX1 binding

The experiments described above suggest that the TFs AP-1 and RUNX1 pay a key role in mediating the resistance of FLT3-ITD+ AML to FLT3 inhibitors. Our ChIP experiments show that (i) RUNX1 and AP-1 binding were lost after FLT3i and (ii) that AP-1 binding was required for RUNX1 to bind at multiple sites. We therefore examined the role of RUNX1 and its connection to signaling processes in more detail. Previous studies had shown that RUNX1 cooperates with FLT3-ITD to generate AML in mice^18^ and that various signaling pathways are required to stabilize the protein.^19^ This was also the case here where RUNX1 protein and FLT3 phosphorylation were lost after FLT3i treatment (Figures 3D and 3E). We next employed two approaches to confirm that RUNX1 itself was required for the growth of FLT3-ITD AML cells. First, we demonstrated that the colony-forming ability of FLT3-ITD+ primary cells was suppressed by RNAi-mediated knock-down of RUNX1 (Figures 3F and S3E). Secondly, we employed AI-14-91 as an inhibitor of RUNX1 binding, which acts by blocking RUNX-CBFβ dimerization (CBFβi)),^20^^,^^21^ to show that RUNX1 inhibition could substitute for FLT3i to block the growth of MV4-11 cells (Figure S4A). Furthermore, AI-14-91 retained the ability to suppress the growth of ITD15 cells that had developed resistance to the FLT3i Quizartinib (Figure S4A). Finally, Gilteritinib and AI-14-91 appear to act on the same pathway, as we found no evidence of synergy of these two inhibitors when employed together (Figures S4B–S4D), again showing the link between FLT3-signalling and the AP-1/RUNX1 axis.

**Figure 4:**
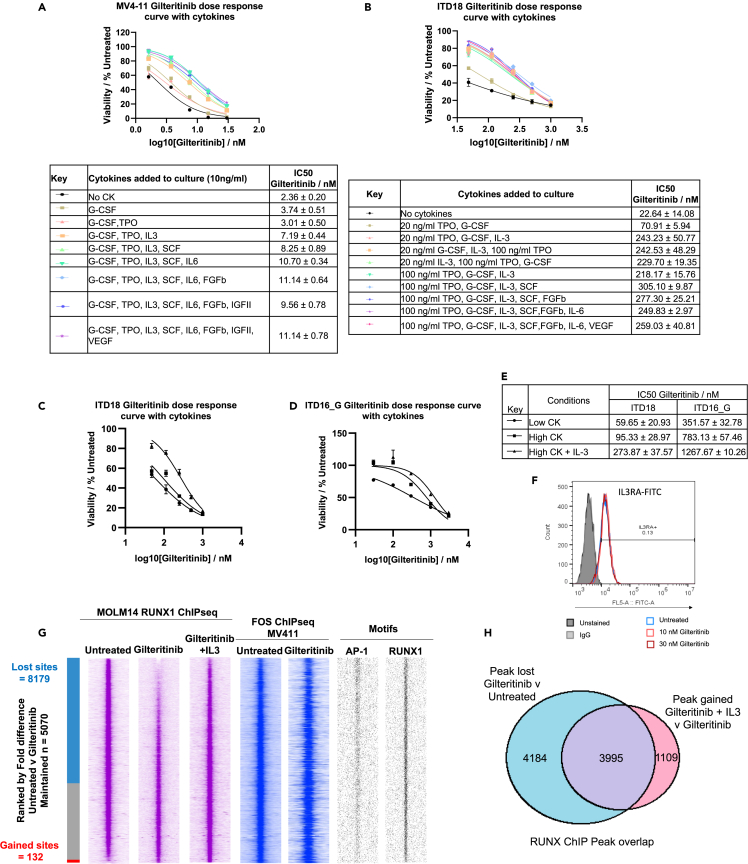
Cytokines mediate FLT3i resistance and restore RUNX binding (A and B) Dose-response curves depicting the viability of MV4-11 (A) and ITD18 (B) cells treated with Gilteritinib in the presence of increasing complexities of cytokines, tables show IC50 values ± standard deviation (n = 3). (C and D) Dose-response curves depicting the viability of primary cells from patients ITD18 (C) and ITD16 (D) relapse samples after FLT3i treated with Gilteritinib cultured in low, high or high+IL-3 cytokines. (E) Table of IC50 values ± standard deviation (n = 3) from figure C and D. (F) Histogram from flow cytometry data of IL3RA surface expression in MOLM14 cells treated with gilteritinib as compared to untreated, and an IgG control. (G) Density plots from ChIP-seq experiments ranked by fold difference in RUNX1 ChIP peak height between Untreated MOLM14 cells and MOLM14s treated with Gilteritinib. RUNX1 ChIP from MOLM14 (pink) and FOS ChIP from MV4-11 (blue) are shown in addition to AP-1 and RUNX1 motif distribution (black). (H) Venn diagram showing the overlap of RUNX1 ChIP peaks which are 2--fold decreased in height after Gilteritinib treatment and those 2-fold higher in Gilteritinib treated MOLM14 cells with IL-3 added.

Because FLT3i treatment caused the up-regulation of multiple signaling pathway genes, we hypothesized that this phenomenon would render AML cells responsive to cytokine stimulation and thus confer resistance to FLT3 inhibition. We therefore assayed the Gilteritinib sensitivity of MV4-11 (Figure 4A) and primary FLT3-ITD cells (ITD18, ITD15, ITD16_G, ITD19) (Figures 4B and S5A) in culture in the presence of cumulative addition of multiple cytokines. In both cases, resistance to FLT3i increased as the complexity of the cytokine mixture in the media increased, with the largest increase in resistance occurring when IL-3 was added. IL-3 conferred resistance to Gilteritinib in MV4-11 and MOLM14 in the absence of other cytokines (Figure S5B), both decreasing cell death, determined by Annexin V and increasing proliferation (Figures S5C–S5F). To further test this notion, we assayed Gilteritinib sensitivity in ITD18 and the FLT3i relapse sample of ITD18 and ITD16_G in the presence of low cytokines (20 ng/mL TPO, G-CSF), high cytokines (100 ng/mL TPO, G-CSF, SCF, FGFII, IL-6, and VEGF) and high cytokines with 100 ng/mL IL-3 (Figures 4C and 4D). Unsurprisingly, the FLT3i IC50 of ITD16 drug-resistant relapse cells (ITD16_G) was an order of magnitude higher than that of ITD18 (Figure 4E). Whilst in both samples, the addition of higher concentrations and complexity of cytokines increased the IC50 of Gilteritinib, the addition of IL-3 to the media increased resistance further. This observation was corroborated in MV4-11 cells (Figure S5G). These results indicate that multiple different cytokines can confer resistance to FLT3 inhibitors in primary cells, although in FLT3-ITD cell lines and some patient samples, IL-3 appears to be substantially responsible, which could be due to the presence of the cell surface receptor of FLT3-ITD+ cell lines (Figure 4F). Interestingly, in patient ITD19 (Figure S5A), FGFII rather than IL-3 was responsible for the log shift in Gilteritinib sensitivity. This resistance due to FGFII has been observed previously and has been shown not to be due to the restoration of STAT5 phosphorylation but due to ERK phosphorylation,^12^ which indicates that targeting the IL-3 receptor alone would not be sufficient to overcome FLT3 inhibitor resistance in all patients.

The observation of the up-regulation of multiple signaling pathways after treatment with FLT3i raised the possibility that FLT3-ITD AML cells could be sensitized to signals from external cytokines, thereby leading to the continued activation of signaling pathways that activate genes via AP-1 and RUNX1. To further investigate this model, we tested the ability of IL-3 to compensate for loss of FLT3 signaling by restoring components of the growth-promoting GRN in MOLM14 cells treated with FLT3i. ATAC-Seq experiments showed a shift in the open chromatin profile in response to FLT3i treatment, which was partially reversed by IL-3 (Figure S6A). To examine the effect of IL-3 on RUNX1 binding, we performed RUNX1 ChIP-seq and RNA-seq on MOLM14 cells treated with 10 nM Gilteritinib in the presence and absence of 10 ng/mL IL-3. The presence of IL-3 in the culture media restored most, but not all, global RUNX1 DNA binding with a strong overlap with FOS-bound regions (Figures 4G and 4H, for quantification see Figure S6B). Conversely, FLT3i led to a reduction of a proportion of FOS peaks overlapping with RUNX1 binding sites (Figure S6C).

**Figure 5:**
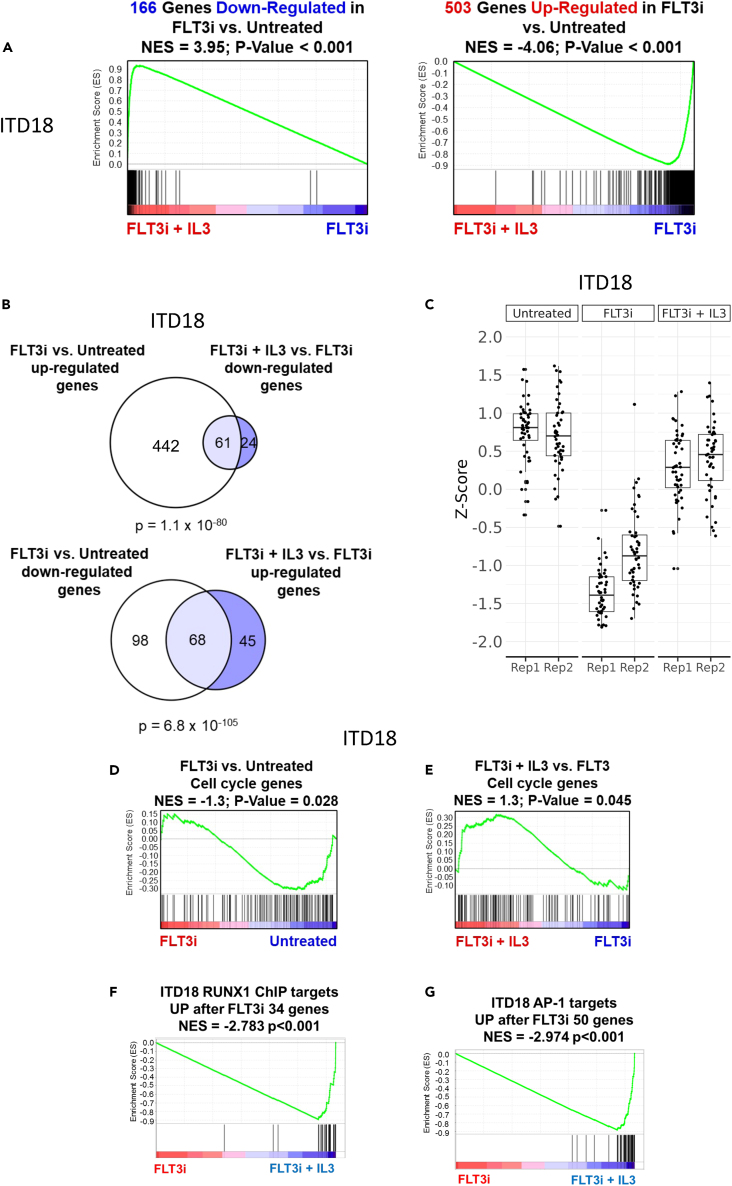
IL-3 supresses the genomic and transcriptomic effects of FLT3 inhibition (A) Gene set enrichment analysis of genes downregulated (left) or upregulated (right) in Gilteritinib treated ITD18 primary cells ranked by the fold change between Gilteritinib treated samples with and without IL-3. (B) Venn diagrams showing the overlap of 2-fold upregulated genes after Gilteritinib treatment compared to untreated, and 2-fold downregulated genes in Gilteritinib treated cells with IL-3 (above) and 2-fold downregulated genes after Gilteritinib treatment compared to untreated, and >2-fold upregulated genes in Gilteritinib treated cells with IL-3 (above) in ITD18 primary cells, p-values were cacluated using a hypergeometric test. (C) Box and whisker plot of RNA expression of cell cycle genes in ITD18 cells with Gilteritinib treatment in the presence or absence of IL-3. (D and E) Gene set enrichment analysis of cell cycle genes in Gilteritinib treated cells vs. untreated (D) and Gilteritinib treated cells in the presence and absence of IL-3. (F and G) Gene set enrichment analyses of RUNX1 (F) and AP-1 (G) target genes in FLT3i cells vs. cells treated with FLT3i plus IL-3.

In parallel with the above changes in TF binding and chromatin accessibility, the addition of IL-3 to FLT3i-treated AML cells substantially reversed the changes in the expression of FLT3i-sensitive genes. Genes inhibited by FLT3i were reactivated by IL-3, and genes activated by FLT3i were repressed after IL-3 addition (Figures 5A and 5B and S6D–S6G). This reciprocal effect was especially apparent when examining cell cycle genes which were downregulated in FLT3i-treated cells. IL-3 restored their expression and cellular growth (Figures 5C, 5D, S6H, and S6I). Moreover, IL-3 prevented the up-regulation of RUNX1 ChIP and AP-1 module targets by FLT3i treatment in both primary cells and cell lines (Figures 5F, 5G, and S6J). In summary, our work presents conclusive evidence that AP-1 and RUNX1 and the response of its target genes sit at the heart of the ability of specific cytokines such as IL-3 to mediate FLT3i resistance.

### Targeting the AP-1 - RUNX1 axis with pan-RAS inhibitors abrogates cytokine-mediated resistance

**Figure 6:**
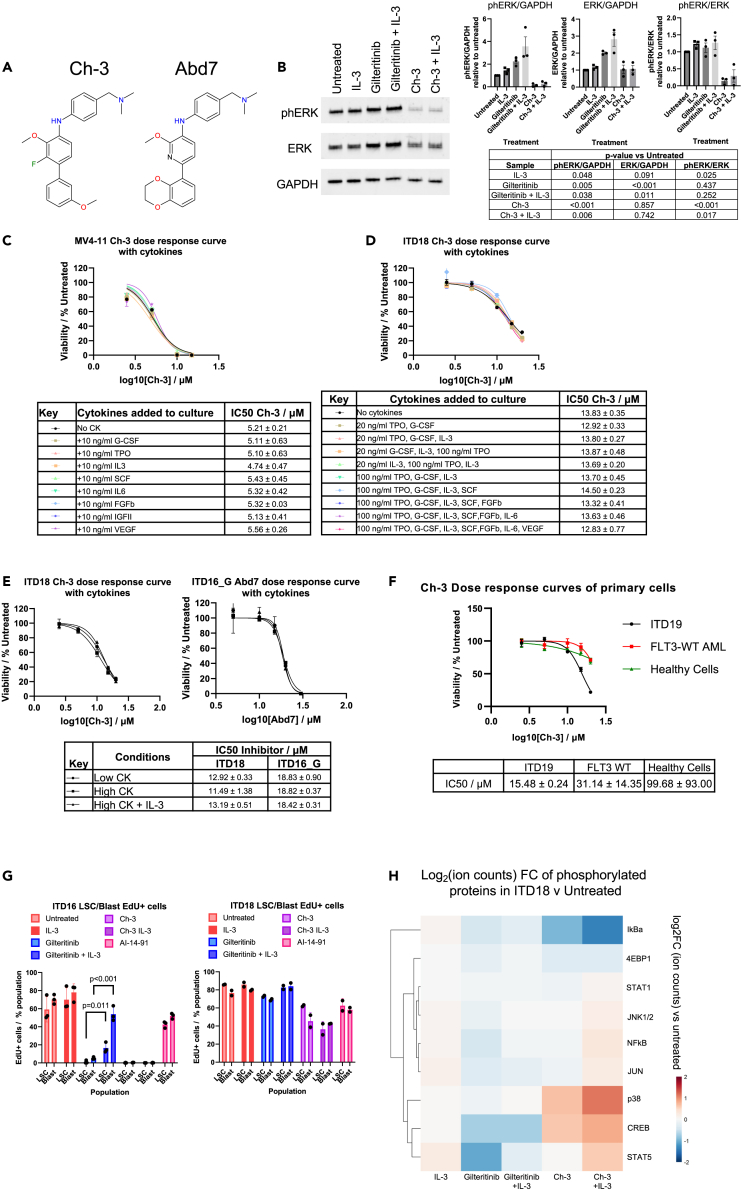
Pharmaceutical inhibition of RAS signaling is unaffected by cytokine treatment (A) Chemical structures of RAS small molecule inhibitors. (B) Western blot of extracts from MOLM14 cells treated with 10 nM Gilteritinib or 15 μM Ch-3 in the presence and absence of 10 ng/mL IL-3. A representative western blot and densitometry of signals from phospho-ERK, ERK and GAPDH are shown (n = 3), error bars show standard deviation, p values of significance vs. the untreated sample were calculated using Student’s t test are shown in the table. (C and D) Dose-response curve depicting the viability of MV4-11 cells (C) and ITD18 primary cells (D) treated with Ch-3 cultured in the presence of various mixtures of cytokines at 10 ng/mL. Tables of IC50 ± standard deviation (n = 3) are shown in bottom panels. (E) Dose-response curves depicting the viability of primary cells ITD18 and ITD16 relapse sample after FLT3i treated with RAS inhibitors in cultures with low, high or high+IL-3 concentrations. The table of IC50s ± standard deviation (n = 3) is included in the bottom panel. (F) Dose-response curves depicting the viability of FLT3-ITD+ AML (ITD19), FLT3 WT AML and heathy CD34^+^ cells treated with Ch-3. The table of IC50 ± standard deviation (n = 3) is shown. (G) Histogram of EDU+ cells from the ITD16_G (n = 3) and ITD18 (n = 2) LSC or Blast populations treated with Gilteritinib or Ch-3 in the presence or absence of 100 ng/mL IL-3. Significant differences are indicated by p values calculated using Student’s t test comparing populations treated with and without IL-3, error bars show standard deviation. (H) Relative signal of phosphorylated signaling proteins detected by CYTOF in ITD18 cells treated with 100 nM Gilteritinib or 20 μM Ch-3 in the presence or absence of 100 ng/mL IL-3. All IC50 values show the mean IC50 ± standard deviation (n = 3).

In order to overcome the FLT3i-resistance that develops in AML relapse cells we aimed to target a point in a signaling pathway where multiple cytokines and signaling mutations converge. A logical candidate downstream of FLT3 and IL-3, and upstream of AP-1 is RAS, which is especially appropriate given that in relapse it is often mutated. To this end, we utilized pan-RAS inhibitors (Figure 6A) which were shown to bind to all RAS isoforms. The compounds were developed as surrogates of the antibody paratope using competition screening.^20^ These compounds inhibit RAS signaling by binding near the switch 1 and switch 2 domains and interfere with the interaction of RAS with its downstream effectors.^22^^,^^23^ Furthermore, they bind to activated wild-type RAS, as it is also found in patients with FLT3-ITD who display constitutively signaling through MAPK as well as mutant RAS, since the similar binding pocket is found in all the RAS isoforms.^23^ We tested the Ch-3 small molecule pan-RAS inhibitor in MOLM14 and FLT3-ITD+ primary cells (ITD15 and ITD16_G) and observed a decrease in ERK phosphorylation (Figures 6B and S7A). We then repeated the cytokine rescue experiments in the presence of the Ch-3 which completely blocked cytokine-mediated resistance (Figures 6C, 6D, S7C, and S7D). This result was also observed with both Ch-3 in the MV4-11 and MOLM14 cell line and in cells from patient ITD18 and ITD19, and with the older generation RAS inhibitor Abd7 in the ITD16 relapse sample after Gilteritinib treatment (Figure 6E, right panel), as well as ITD15 (Figure S7D). Ch-3 also decreased colony-forming ability in FLT3-ITD+ primary cells (Figure S7B). FLT3-ITD+ AML cells showed greater sensitivity to the pan-RAS inhibitor than either CD34^+^ cells, or an AML with wild-type FLT3 plus a MOZ::TIF rearrangement (Figure 6F).

We next investigated whether Ch-3 was also able to suppress the growth of the leukemic stem cells (LSCs) which are generally quiescent but are thought to be responsible for the relapse of AML following chemotherapy.^24^^,^^25^ To this end, we determined whether the FLT3 inhibitor and pan RAS inhibitor differentially affect the growth of LSC and blast cells of primary FLT3-ITD+ AML cells (ITD16_G and ITD18) in culture. In this experiment, CD34+/CD38- (LSC) and CD34+/CD38+ (blast) cells were sorted by FACS and stained with two different membrane dyes prior to culture in high cytokine conditions with or without 100 ng/mL IL-3 and 100 nM Gilteritinib, 20 μM Ch-3 or 10 μM AI-14-91 (Figure S7E). LSCs from several patients with FLT3-ITD previously sorted with this strategy formed colonies *in vitro*, whilst the blast cells did not (data not shown). EdU was included in the culture media to measure the growth rate of the two populations. After 6 days of culture, the cells were stained with FITC-linked antibodies targeting EdU and analyzed by FACS, whereby the membrane dyes distinguished whether cells were derived from the LSCs or blasts. While the FLT3i-treated cells showed a reduction in growth in both LSC and blast-derived populations, the addition of IL-3 rescued the growth of both populations (Figure 6G). The addition of IL-3 did not rescue growth in either population in cells treated with the RAS inhibitor. Targeting RUNX1 with the AI-14-91 also reduced the growth of both populations. In ITD16_G, Gilteritinib treatment significantly reduced the ratio of LSCs to blasts, which was rescued by IL-3. However (Figure S7F), we did not observe this in sample ITD18, although note that the variation between samples was larger in the treatments where the growth rate was reduced.

To examine, which signaling molecules were affected by the different treatments, we performed mass cytometry (CyTOF) analysis of multiple phosphorylated signaling proteins on both ITD18 in high cytokine conditions and MOLM14 cells with and without IL-3 and FLT3 or RAS inhibition (Figures 6H and S7G). In both experiments, FLT3i treatment caused a decrease in STAT5 phosphorylation, which was rescued by the addition of IL-3. IL-3 also increased STAT5 signaling in the Ch-3 treated cells, indicating that this is no longer sufficient to rescue the phenotype mediated by this inhibitor. The RAS inhibitor caused a decrease in IkB phosphorylation and an increase in NF-kB phosphorylation, although this was more pronounced in the MOLM14 cells. ERK phosphorylation was heavily reduced by Ch-3 in the MOLM14 cells, but the signal was not detectable in the ITD18 sample. In the primary cells but not in MOLM14 cells, CREB and p38 showed increased phosphorylation, which could indicate that in the high cytokine conditions, ITD18 cells are attempting to rewire signaling through p38.

**Figure 7:**
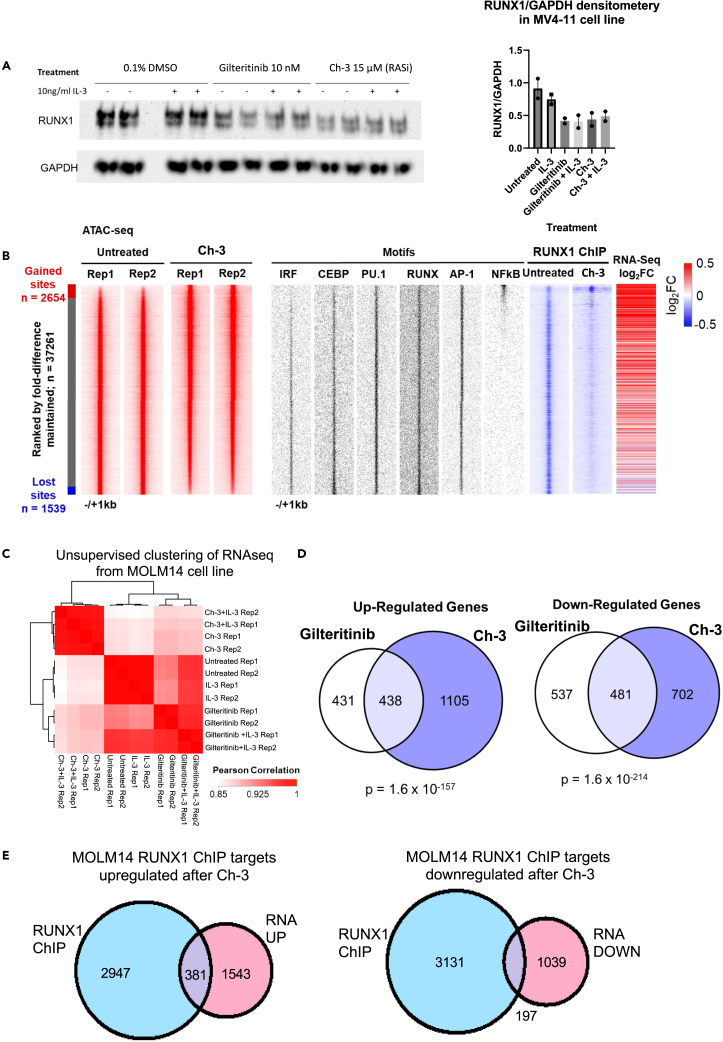
RAS inhibition destabilises RUNX chromatin binding and causes transcriptomic changes irrespective of the presence of IL-3 (A) Western blot of RUNX1 protein expression in MOLM14 cells treated with 10 nM Gilteritinib or 15 μM Ch-3 in the presence or absence of IL-3. The histogram shows densitometry analysis (n = 2) with error bars showing standard deviation. (B) Genome wide data from MOLM14 cells treated with Ch-3. Density plots of ATAC-Seq data (red) are ranked by the fold change of ATAC peak height between untreated and Ch-3 treated cells. The distribution of enriched Motifs of the indicated TF families are plotted along-side (black) as is the RUNX1 ChIP-Seq peak tag-count in untreated cells and those with RAS inhibitor expression. The fold change of expression of genes linked to sites is also shown (far right panel). (C) Unsupervised clustering of the Pearson correlation values of RNA-seq data from MOLM14 cells treated with Gilteritinib or Ch-3 in the presence or absence of IL-3. (D) Venn diagram showing overlap of genes 2-fold upregulated (above) or downregulated (later in discussion) in MOLM14 after Gilteritinib or Ch-3 treatment. (E) Venn diagrams showing overlap of RUNX1 target genes upregulated (left) or downregulated (right) after RAS inhibitor treatment in MOLM14 cells.

To be able to measure the binding of transcription factors in the presence and absence of RAS, we constructed a MOLM14 cell expressing a Doxycycline-inducible RAS inhibitor peptide^23^ from a lentiviral vector. Similar to the FLT3 inhibitor, RAS inhibition reduced the level of RUNX1 protein (Figure 7A) and prevented the DNA binding of RUNX1 as measured by ChIP analysis in MOLM14 cells after DOX induction (Figures 7B and S8A). However, note that IL-3 did not bring RUNX1 levels up to that of untreated cells after FLT3i, suggesting that not the protein but signaling that regulates RUNX1 activity is important as reported previously.^19^ The binding sites lost in the RAS inhibitor-treated cells and the FLT3i-treated cells were largely the same (Figures S8B and S8C) and also correlated with those found in the MV4-11 FOS ChIP. ATAC peaks that were lost after RAS inhibition were enriched for DNA motifs for the myeloid regulators C/EBP, PU.1, and IRF, suggesting that the cells shifted to a more immature state. Interestingly, NF-kB motifs were strongly enriched, specifically in the gained ATAC sites which correlates with the increased phosphorylation of NF-kB observed in these samples (Figure S7G). In this respect, it is interesting to note that a reduction of NF-kB-mediated inflammatory signaling was shown to be required for the growth of MV4-11 cells.^26^ In addition, in both primary cells and the cell line, RAS inhibition caused retained dephosphorylation of the translation inhibitor 4EBP1,^27^ thereby inactivating it, increasing the anti-tumour effect of Ch-3 and explaining the reduction in RUNX1 protein levels.

We next performed RNA-Seq analysis of MOLM14 cells treated with 15 μM Ch-3 in the presence and absence of 10 ng/mL IL-3 followed by the unsupervised clustering of the Pearson correlation values (Figure 7C). This clustering shows that, while the addition of IL-3 to FLT3i-treated MOLM14 cells increases the correlation with the untreated cells, the cytokine has no such effect on RAS inhibitor-treated cells, which was confirmed by GSEA analysis (Figure S8D). The 2-fold deregulated genes after Ch-3 treatment overlapped with those deregulated after FLT3i treatment (50% upregulated, 47% downregulated) indicating that the inhibition of RAS mimics the effect of inhibiting FLT3-ITD in these cells (Figure 7D). In ITD18 primary cells, the presence of 100 ng/mL IL-3 also did not suppress the effects of the RAS inhibitor on gene expression (Figures S8E and S8F), although the overlap with FLT3i deregulated genes was less substantial (Figure S8G). Finally, we compared the 2-fold deregulated genes after RAS inhibitor treatment with the RUNX1 ChIP and observed that 20% upregulated and 16% downregulated genes were direct targets of RUNX1 (Figure 7E).

**Figure 8:**
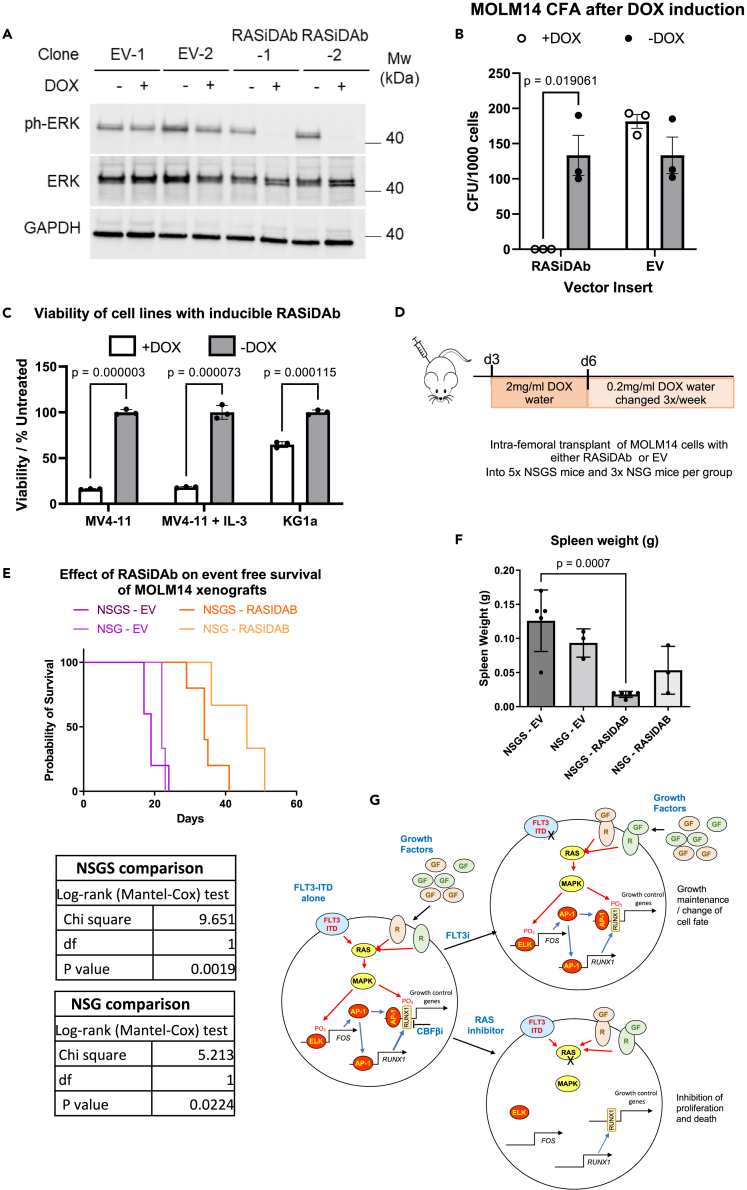
FLT3-ITD+ cells expressing an inducible RAS inhibitory antibody fragment slow progression of AML *in vivo* (A) Western Blot analysis of MOLM14 FLT3-ITD cells transduced with empty vector or RAS iDAb expressing vector with or without DOX induction showing phospho-ERK, ERK and GAPDH. (B) Colony formation assay of MOLM14 cells transduced with empty vector or RAS iDAb expressing vector with or without DOX induction, error bars show standard deviation for (n=3) samples, p values were calculated using Student's t test. (C) Histogram shows the viability of MV4-11 cells and non-FLT3-ITD KG1a cells with the RASiDAb construct after 3 days induction. (D) Design of *in vivo* experiment. (E) Kapplan Meier survival curves of mice xenografted with MOLM14 cells transduced with empty vector or RAS iDAb expressing vector, tables show p values calculated using the log-rank (Mantel-Cox) test. (F) Histogram of spleen sizes in mice engrafted with MOLM14 cells, error bars show standard deviation and p values were calculted using Student's t test (n = 5 for NSGS and n = 3 for NSG). (G) Proposed model for RAS inhibition overcoming cytokine-mediated cell survival. For details see main text.

To determine whether targeting RAS in FLT3-ITD cells might be a viable strategy *in vivo* we employed MOLM14 cells expressing the inducible RAS inhibitory antibody fragment (RASiDAb)^23^ or an empty vector control. After selecting single-cell clones, we confirmed the effectiveness of the RAS inhibition *in vitro* by Western Blot (Figure 8A) and confirmed that the induction of the antibody prevented the colony formation of MOLM14 cells (n = 3 independent clones) (Figure 8B) and reduced growth in the presence and absence of IL-3 to a greater degree in the FLT3-ITD MV4-11 cell line compared to a FLT3-WT KG1a control (Figure 8C). NSGS mice,^28^ which express human IL-3 and NSG mice which do not, were transplanted with MOLM14 cells. Doxycycline induction of the cells was commenced after 3 days to ensure cells were engrafted prior to the induction of the inhibitory antibody fragment. After the first 3 days of 2 mg/mL doxycycline induction, a lower dose of 0.2 mg/mL was maintained for the remainder of the treatment (Figure 8D). Mice engrafted with MOLM14 cells expressing the inhibitory RAS antibody fragment showed significantly increased survival in both strains of mice compared to the empty vector control (Figure 8E). Although the NSGS mice with the RAS inhibitor expressing cells survived less long than their NSG counterparts, their spleen weight was significantly reduced in comparison to mice carrying cells with the empty vector (Figure 8F).

Taken together, our study shows that (i) the AP-1- RUNX1 axis is essential for mediating drug resistance in patients treated with FLT3i and (ii) that targeting this pathway at multiple points provides a viable way to counteract drug resistance and increase survival.

## Discussion

In our study, we addressed the general question of how cancer cells that rely on chronic growth factor signaling bypass targeted inhibition by examining the molecular basis of drug resistance against FLT3 inhibitors in AML. Figure 8G depicts a model of the molecular details of how the FLT3-ITD AML gene regulatory network is rewired in response to FLT3 inhibition. Using a dominant-negative FOS peptide, we previously showed that the AP-1 family of signaling-responsive TFs is essential for FLT3-ITD AML development *in vivo*.^1^ AP-1 is part of a highly complex transcriptional circuitry that links chronic FLT3-ITD MAPK signaling to the expression of multiple cell cycle regulator and growth factor receptor genes.^14^^,^^29^^,^^30^ Our data show that the other part of this circuitry consists of RUNX1. RUNX1 sits directly downstream of FLT3-ITD signaling and protein levels are reduced after the inhibition of FLT3 through the removal of phosphate residues on tyrosines in the inhibitory domain^18^ and, as we show here, by activating a translation block (4EBP1). Posttranslational modifications (PTMs) of RUNX1 are important regulators of its activity.^19^ RUNX1 undergoes multiple different PTMs through different signaling pathways, which regulate the interactions with corepressors/coactivators thus mediating both activating and repressive activities.^19^^,^^31^^,^^32^^,^^33^ Importantly, many growth factor receptor genes, such as *IL-3RA* are themselves targets of RUNX1.^34^ IL-3 signaling and RUNX1 activity appear to be closely linked as IL-3 can rescue RUNX1 knockout in HPSCs and leukemic cells, and loss of RUNX1 increases the expression of *IL3RA*^35^ which we also see upregulated after FLT3 inhibition. IL-3 signals through both JAK/STAT and MAPK^36^ and both pathways have been shown to interact with RUNX1. STAT5 can prevent the nuclear localization of RUNX1 and supresses the activity of both TFs.^37^ Whilst targeting the JAK-STAT pathway with Ruxolitinib has been reported as a mechanism by which IL-3 mediated resistance can be addressed in FLT3-ITD AML^11^ this treatment will not prevent the IL-3 mediated activation of ERK and RUNX1, which potentiates the transactivation activity of RUNX1.^38^ This interaction would explain the IL-3 mediated rescue of RUNX1 binding observed in our ChIP-seq experiments after FLT3 inhibition and targeting MAPK signaling rather than JAK-STAT would prevent this interaction.

However, RUNX1 alone is not the whole story. Both AP-1 and RUNX1 are essential for signaling-mediated growth regulation in FLT3-ITD AML cells and bind in close proximity to each other in chromatin, thus integrating multiple signaling inputs. Their binding at such sites is signaling-dependent since sites where FOS was lost after FLT3 inhibition lost RUNX1 binding as well, indicating that the interplay between the two TFs was disrupted. Moreover, the inhibition of AP-1 binding by using a dnFOS peptide is sufficient to abrogate RUNX1 binding at thousands of sites as well. Here AML cells appear to hijack a normal control mechanism that links RUNX1-mediated cytokine gene and cell cycle gene regulation to growth factor signaling. Healthy cells are much less dependent on continuous signaling as their proliferation is subject to strong demand-driven feedback control. An important consequence of this aberrant circuitry is therefore that it gives AML cells the flexibility to maintain growth in response to multiple growth factor environments.

FLT3i treatment (Figure 8G upper right panel) leads to the loss/redistribution of AP-1 and RUNX1 binding. The consequence is an upregulation of genes associated with signaling as observed in our data and reported previously.^39^^,^^40^ It was previously shown that patients unresponsive to Gilteritinib treatment upregulate signaling genes rapidly after treatment, compared to those which showed a response.^41^ Our patient and *in vitro* data suggest that in unresponsive patients the GRN is being or has already been rewired toward an increased reliance on AP-1 mediated signaling. We hypothesise that this response primes the cells to receive extracellular stimulation by other growth factors to restore proliferation and prevent cell death due to the loss of FLT3-ITD signaling and is the cause for relapse. However, the mechanism by which up-regulation occurs is still unclear and may involve post-transcriptional mechanisms such as RNA stability regulation in response to the block in cell cycle and translation as it is seen with cytokine genes.^42^ The pan-RAS inhibitor targets the entire AP-1 - RUNX1 axis and bypasses the rescue of growth via external growth factors and cytokine-mediated resistance (Figure 8F, lower right panel). FLT3-ITD AML are particularly sensitive to this inhibitor as, due to the chronic activation of FLT3, RAS in is a constitutively active conformation. This idea is supported our observation that the FLT3-ITD AML cells were more sensitive to RAS inhibition than healthy CD34 cells or AML without a MAPK activating mutation in culture. By targeting the RAS family, we avoid the redundancy of other members of the signaling cascade, which allows cells to rewire to bypass inhibition.

Cytokine-mediated resistance to FLT3 inhibitors has been previously described in connection with the bone marrow niche,^11^^,^^12^ but suggested treatments often target JAK STAT signaling^11^^,^^41^ or individual surface receptors responsible for resistance such as FGFR1.^12^ Due to the upregulation of multiple different signaling genes in FLT3-ITD AML after FLT3 inhibition these treatments are still vulnerable to cytokine-mediated resistance from other factors, also due to the heterogeneity of responses to FLT3 inhibitors in cell lines and patient samples. In conclusion, by carefully interrogating drug response at the systems level, we uncovered that through the direct targeting of RUNX1 itself and RAS family signaling a key mechanism of resistance to targeted therapy in FLT3-ITD AML can be overcome. We believe that such studies are key to find treatments that block cancer resurgence.

### Limitations of the study

We are aware that there are limitations to this study. Patient material to conduct gene regulatory network analysis on matched samples before and after FLT3 inhibitor treatment is difficult to obtain in a quality where genomic profiling will be informative. We were unable to perform the FOS ChIP experiment in the MOLM14 cell line and as a result have compared the MOLM14 and MV4-11 ChIP-seq experiments. However, due to the similarities in response to FLT3 inhibitors we do not think this has influenced our conclusions. It is possible that the xenotransplantation experiments do not fully reflect the human situation. Human bone marrow organoids were recently described, which could alleviate this problem.

## STAR★Methods

### Key resources table

**Table:** 

REAGENT or RESOURCE	SOURCE	IDENTIFIER
**Antibodies**		

Phospho-FLT3 antibody (Tyr589/591) (30D4) Antibody	Cell Signaling Technology	Cat# 3464; RRID: AB_2107051
Phospho p44/42 MAPK (Erk1/2) (Thr202/204) Antibody	Cell Signaling Technology	Cat# 9101; RRID: AB_331646
p44/42 MAPK (Erk1/2) Antibody	Cell Signaling Technology	Cat# 9102; RRID: AB_330744
AML1 Antibody	Cell Signaling Technology	Cat# 4334; RRID: AB_2184099
Anti-GAPDH Antibody [6C5]	Abcam	Cat# ab8245; RRID: AB_2107448
Anti-RUNX1/AML antibody	Abcam	Cat# ab23980; RRID: AB_2184205
Anti-rabbit IgG HRP-linked antibody	Cell Signaling Technology	Cat# 7074; RRID: AB_2099233
Anti-mouse IgG HRP-linked antibody	Cell Signaling Technology	Cat# 7076; RRID: AB_330924
Anti-goat IgG HRP-linked antibody	Jackson ImmunoResearch	Cat# 115-035-062; RRID: AB_2338504
cFOS antibody	Thermo Fisher Scientific	Cat# MA5-15055; RRID: AB_10984728
CD117 Antibody, anti-human, APC, REAfinity	Miltenyi Biotec	Cat# 130-111-593; RRID: AB_2654579
CD123 Antibody, anti-human, FITC, REAfinity	Miltenyi Biotec	Cat# 130-115-263; RRID: AB_2726967
REA Control Antibody (S), human IgG1, FITC, REAfinity	Miltenyi Biotec	Cat# 130-113-437; RRID: AB_2733689
REA-Control (I)-APC	Miltenyi Biotec	Cat# 130-104-615; RRID: AB_2661679
CD34 PE-Cy7 CE	BD Biosciences	Cat# 348811; RRID: AB_2868855
CD38 V450 RUO	BD Biosciences	Cat# 646851; RRID: AB_1937282
Lineage Cocktail 1 (Lin 1) FITC	BD Biosciences	Cat# 340546; RRID: AB_400053
CD184 (CXCR4) Antibody, anti-human, PE, REAfinity	Miltenyi Biotec	Cat# 130-117-354, RRID: AB_2733780
Anti-IL10RA Antibody (FITC)	CUSABIO	Cat# CSB-PA621688EC01HU; RRID: AB_3086808
CD217 (IL17RA) Antibody, anti-human, APC, REAfinity	Miltenyi Biotec	Cat# 130-127-293; RRID: AB_2904838
Mouse anti-human CD45-89Y	Standard BioTools	Cat# 3089003; RRID: AB_2661851
Mouse anti-human CD34-148Nd	Standard BioTools	Cat# 3148001B; RRID: AB_2810243
Mouse anti-human CD38-167Er	Standard BioTools	Cat# 3167001B; RRID: AB_2802110
Mouse anti-human Ki-67-172Yb	Standard BioTools	Cat# 3172024B; RRID: AB_2858243
Mouse anti-human CD117	BioLegend	Cat# 313202; RRID: AB_314981
Rabbit anti-pSTAT1 (Y701)-153Eu	Standard BioTools	Cat# 3153003A; RRID: AB_2811248
Mouse anti-pSTAT3 (Y705)-158Gd	Standard BioTools	Cat# 3158005A; RRID: AB_2811100
Mouse anti-pSTAT5 (Y694)-150Nd	Standard BioTools	Cat# 3150005A; RRID: AB_2744690
Mouse anti-pS6 (S235/S236)-175Lu	Standard BioTools	Cat# 3175009A; RRID: AB_2811251
Rabbit anti-pCREB (S133)-176Yb	Standard BioTools	Cat# 3176005A; RRID: AB_2934290
Mouse anti-pNFκB-p65 (S529)-166Er	Standard BioTools	Cat# 3166006A; RRID: AB_2847867
Mouse anti-IκBα-164Dy	Standard BioTools	Cat# 3164004A; RRID: AB_2811249
Rabbit anti-p4E-BP1 (T37/T46)-149Sm	Standard BioTools	Cat# 3149005A; RRID: AB_2847866
Rabbit anti-p-Jnk1/Jnk2 (T183/Y185)	ThermoFisher	Cat# 700031; RRID: AB_2532273
Rabbit anti-p-cJun (S243)	ThermoFisher	Cat# PA5-104747; RRID: AB_2816220
Anti-human cleaved PARP (F21-852)-143Nd	Standard BioTools	Cat# 3143011A; RRID: AB_2927562
Anti-pAkt [S473] (D9E)-152Sm	Standard BioTools	Cat# 3152005A; RRID: AB_2811246
Anti-pERK1/2 [T202/Y204] (D13.14.4E)-171Yb	Standard BioTools	Cat# 3171010A; RRID: AB_2811250

**Bacterial and virus strains**		

NEB Stable Competent E Coli	New England Biolabs	C3040

**Chemicals, peptides, and recombinant proteins**		

BD Pharmingen 7-AAD	BD Biosciences	559925
7-Aminoactinomycin D	Sigma-Aldrich	A9400-1MG
Recombinant human TPO	PeproTech	300–18
Recombinant human IL-3	PeproTech	200–03
Recombinant human G-CSF	PeproTech	300–23
Recombinant human SCF	PeproTech	300–07
Recombinant human IL-6	PeproTech	200–06
Recombinant human FGF-basic	PeproTech	100-18B
Recombinant human VEGF 165	PeproTech	100–20
Recombinant human IGF-II	PeproTech	100–12
FBS Qualified	Gibco	10270–106
HEPES solution 1M pH7.4	Sigma	H0887
Penicillin/Streptomycin	Gibco	15070–063
MACS BSA Stock Solution	Miltenyi Biotech	130-091-376
RPMI 1640 Medium	Sigma Aldrich	R8758
Dulbeccos Modified Eagles Medium	Sigma Aldrich	D6546
UM729	StemCell Technologies	72332
StemRegenin 1	StemCell Technologies	72344
L-Glutamine	Gibco	25030081
Calcium Chloride dihydrate	Sigma Aldrich	C3306
Sodium Chloride	Acros Organics	207790050
HEPES	Sigma Aldrich	H3375
Sodium phosphate dibasic	Sigma Aldrich	S3397
Polybrene	Sigma Aldrich	TR-1003-G
Doxycycline	Sigma Aldrich	D5207
Phosphate Buffered Saline	Merck	806552
Nusieve 3:1 Agarose	Lonza	50090
Di(N-succinimidyl) glutarate	Sigma Aldrich	80424
Ampure XP SPRI Reagent	Beckman Coulter	A63881
Methocult Express	StemCell Technologies	04437
Methocult H4100	StemCell Technologies	04100
Laemmli buffer	Bio-Rad	1610747
Enhanced chemiluminescent reagent	Cytiva	RPN2134
Tris-HCl	Fisher Bioreagents	BP153-1
Tween 20	Sigma Aldrich	P2287
Restore Stripping Buffer	Thermo Scientific	21059
β-mercaptoethanol	Sigma Aldrich	M3148
Hydrocortisone	Sigma Aldrich	H0888
Gilteritinib	Selleckchem	S7754
Ch-3	Cruz-Migoni et al.^23^	N/A
Abd7	Cruz-Migoni et al.^23^	N/A
AI-14-91	Illendula et al.^20^	N/A
DMSO	Merck	D2650
Trypan Blue	Merck	T8154
Magnesium chloride	Fisher Scientific	M/0600/53
Tn5 transposase enzyme and TD buffer	Illumina	15027865/6
Nonidet P-40	BDH Laboratory Supplies	56009
Digitonin	Promega	G944A
NEBNext® High-Fidelity 2X PCR Master Mix	New England Biolabs	M0541S
16% formaldehyde (methanol free)	Thermo Scientific	28906
Triton X-100	Sigma Aldrich	T8787
Glycine	Merck	357002
EDTA	Sigma Aldrich	E5134
EGTA	Sigma Aldrich	E3889
Complete Mini Protease Inhibitor Cocktail	Merck	04693124001
Sodium dodecyl sulfate	Sigma Aldrich	L5750
Glycerol	Fisher Scinetific	G/0650/17
Dynabeads-Protein G	Invitrogen	10004D
Albumin, Acetylated from bovine serum	Merck	B2518
Phosphate citrate buffer tablet	Sigma Aldrich	P4809
Lithium chloride	Sigma Aldrich	L9650
Sodium deoxycholate	Alfa Aesar	B20759
Sodium bicarbonate	Sigma Aldrich	S6297
Lymphoprep	StemCell Technologies	07851
Horse serum, heat inactivated	Gibco	11540636
alpha-MEM	Lonza	12–169
Sodium pyruvate solution	Sigma-Aldrich	S8636
Taq DNA polymerase	Thermo Fisher	EP0401
RNase A	Sigma Aldrich	10109169001
Sodium citrate	Sigma Aldrich	C8532
Iscoves Modified Dulbeccos Medium	Sigma Aldrich	I3390
Superscript II Reverse Transcriptase	Thermo Fisher	18064014
Tris-(2-Carboxyethyl)phosphine, Hydrochloride (TCEP)	Fisher Scientific	10286352

**Critical commercial assays**		

PKH26 Red Fluorescent Cell Linker Kit for General Cell Membrane Labeling	Sigma Aldrich	PKH26GL
CellVue® Claret Far Red Fluorescent Cell Linker Mini Kit for General Membrane Labeling	Sigma-Aldrich	MINCLARET
CD117 MicroBead kit, human	Miltenyi Biotec	130-091-332
CD34 MicroBead kit, human	Miltenyi Biotec	130-046-702
CellTiter-Glo 2.0	Promega	G9241
FITC Annexin V Apoptosis detection kit with 7-AAD	Biolegend	640922
QIAquick Gel Extraction kit	Qiagen	28706
EndoFree Plasmid Maxi Kit	Qiagen	12362
DNeasy Blood and Tissue Kit	Qiagen	69504
NextSeq 500/550 High output v2.5 kit (75 cycles)	Illumina	20024906
NextSeq 500/550 High output v2.5 kit (150 cycles)	Illumina	20024907
Turbo transfer packs	Bio-rad	1704156
Mini PROTEAN TGX Gels	Bio-rad	4561096
QIAquick PCR clean up kit	Qiagen	28006
RNeasy Micro Plus kit	Qiagen	74034
RNeasy Micro kit	Qiagen	74004
NEBnext Ultra II Directional RNA Library Prep Kit for Illumina	New England Biolabs	E7760
NEBNext® rRNA Depletion Kit v2 for	New England Biolabs	E7400
TruSeq RNA Library Preparation Kit	Illumina	RS-122-2001
Kapa Hyper prep kit	Roche	07962363001
High Sensitivity DNA kit	Agilent	5067–4626
Kapa Library Quantification kit	Roche	07960204001
EdU proliferation kit iFluor 488	Abcam	ab219801
MaxPar X8 antibody-labelling kit	Standard BioTools	201300

**Deposited data**		

ATAC-seq, DNaseI-seq, RNA-seq and ChIP-seq data	This paper	GEO: GSE241650

**Experimental models: Cell lines**		

MOLM-14	DMSZ	Cat# ACC777; RRID:CVCL_7916
MV4-11	DMSZ	Cat# ACC102; RRID:CVCL_0064
HEK293T	DMSZ	Cat# ACC635; RRID:CVCL_0063
Kg-1a	DMSZ	Cat# ACC421; RRID:CVCL_1824

**Experimental models: Organisms/strains**		

NOD.Cg-Prkdcscid Il2rg tm1Wjl/SzJ	In house breeding – Newcastle University	N/A
NOD.Cg-Prkdcscid Il2rgtm1Wjl Tg(CMV-IL3,CSF2,KITLG)1Eav/MloySzJ	In house breeding – Newcastle University	N/A

**Oligonucleotides**		

PCR primers	See Table S2 for sequences	N/A
siRUNX1	Dharmacon	J-003926-07
siMM	CCUCGAAUUCGUUCUGAGAAG	Custom

**Software and algorithms**		

GRN construction scripts	https://doi.org/10.5072/zenodo.268	https://github.com/petebio/Gene_regulatory_network_analysis
Prism	Graphpad	v9.4.1
Trimmomatic	Bolger et al.^43^	v0.39
HISAT2	Kim et al.^44^	v2.2.1
featureCounts	Liao et al.^45^	v2.0.1
Limma-Voom	Law et al.^46^	v3.50.3
R	https://www.r-project.org/	v4.1.2
ClueGO	Bindea et al.^47^	v2.5.0
edgeR	Robinson et al.^48^	v3.36.0
Bowtie2	Langmead and Salzberg^49^	v2.2.5
Picard MarkDuplicates	http://broadinstitute.github.io/picard	v2.26.10
MACS2	Zhang et al.^50^	v2.2.7.1
bedtools	Quinlan and Hall^51^	v2.30.0
Homer	Heinz et al.^52^	v4.9.1
Java Treeview	Saldanha^53^	v1.1.6r4
Cytoscape	Shannon et al.^54^	3.10.0
FlowJo	www.flowjo.com	v10.8.1
samtools	Li et al.^55^	v1.12
GSEA software	BROAD Institute^56^	v2.2.4

### Resource availability

#### Lead contact

Further information and requests for resources and reagents should be directed to and will be fulfilled by the lead contact, Constanze Bonifer (c.bonifer@bham.ac.uk).

#### Materials availability

No materials were generated by this study.

#### Data and code availability

• All sequencing data produced as part of this study are available on GEO and are publicly available as of the date of publication under the super series GEO: GSE241650.
• Python scripts used to construct the gene regulatory networks presented in this study, as well as the probability weight matrices for the transcription factor binding motifs and promoter-capture HiC data have been made available on GitHub at https://github.com/petebio/Gene_regulatory_network_analysis and are free to use under an MIT license, https://doi.org/10.5072/zenodo.268, these scripts have been published previously.^14^
• Any additional information required to reanalyse the data reported in this paper is available from the lead contact upon request.

### Experimental model and study participant details

#### Primary sample and PBSC processing

Human tissue was obtained with the required ethical approval from the National Health Service (NHS) National Research Ethics Committee. AML and PBSC samples used in this study were fresh samples obtained with specific consent from the subjects. AML samples were obtained from the Centre for Clinical Haematology, Queen Elizabeth Hospital Birmingham, Birmingham, UK. In the case of patient ITD16, surplus DNA and RNA from diagnostic samples were obtained from the West Midlands Regional Genetics Laboratory, Birmingham Women’s NHS Foundation Trust, Birmingham, UK. Upon receipt mononuclear cells were purified by lymphoprep and samples were further purified using either CD34 or CD117 microbead kits (Miltenyi Biotec). For samples with >92% blast cells purification was not performed. Human Mobilized Peripheral Blood CD34^+^ Cells, used as healthy controls, were purchased from AMS Biotechnology (Europe) Limited. Samples used to generate human mesenchymal stem cells (hMSCs) for co-culture were obtained with consent from subjects from the Centre for Clinical Haematology, Queen Elizabeth Hospital Birmingham, Birmingham, UK. Samples were screened for mutations using the Illumina Trusight myeloid panel of primers, and analysed using algorithms to detect either nucleotide variants using the Genome Analysis Toolkit^57^ or insertions and deletions using Pindel.^58^ Mutations were also screened against the COSMIC database of previously observed mutations. Mutated genes are listed in Table S1.

#### Primary cell culture

Human mesenchymal stem cells were expanded from bone marrow aspirates from patients with normal bone marrow. Briefly, CD34^−^cells were collected from the flow through of the MACS sort using the human CD34 microbead kit. These cells were cultured in alpha-MEM (Lonza) supplemented with 10% fetal calf serum (Gibco), 100 U/mL penicillin/streptomycin and 2 mM L-Glutamine (Gibco) and adherent cells were expanded in culture for 4 weeks prior to freezing for future experiments.

Primary AML cells were defrosted by gradual addition of 5% fetal calf serum (FCS) (Gibco) PBS (Sigma-Aldrich) with 400 μg/mL DNaseI (Roche). Defrosting media was removed by centrifugation and cells were resuspended at 0.3–0.5 × 10^6^ cells/mL and cultured on hMSC feeders in alpha-MEM (Lonza) supplemented with 12.5% fetal calf serum, 12.5% horse serum, 100 U/mL penicillin/streptomycin, 2 mM L-Glutamine (all Gibco), 1 μM hydrocortisone (Merck) and 57.2 μM β-mercaptoethanol (Merck), 20 ng/mL IL-3, G-CSF and TPO (Pepro Tech). Cells were cultured on hMSC feeders for 7 days prior to experiments.

All cells were cultured in an incubator at 37°C with 5% CO_2_.

#### Cell lines

MV4-11 (DMSZ, AC102), MOLM14 (DSMZ, ACC 777) and KG-1a (DMSZ, ACC 421) were cultured in RPMI 1640 supplemented with 10% fetal calf serum, 2 mM L-glutamine and 100 u/mL penicillin/streptomycin (all Gibco) at 37°C with 5% CO_2_. Human embryonic kidney 293T (HEK293T) cells were cultured in DMEM supplemented with 10% FCS, 2 mM L-glutamine, 100 U/mL penicillin, 100 mg/mL streptomycin and 0.11 mg ml–1 sodium pyruvate.

#### Xenograft mouse studies

Mouse studies were carried out in accordance with UK Animals (Scientific Procedures) Act, 1986 under project licence P74687DB5. Ethical approval for the study was provided by Newcastle University animal ethical review body (AWERB). Mice were housed in specific pathogen free conditions in individually ventilated cages with sterile bedding, water and diet (Irradiated TekladTM Global 19% Protein Extruded Rodent Diet 2919, Inotiv). All procedures and handling were performed aseptically in a laminar flow hood.

MOLM-14 cells (acquired from DSMZ ACC-777), engineered to express either RASIDAB or empty vector control in the presence of doxycycline, were injected intravenously into the tail vein; 50,000 cells per mouse in 50 μL of RPMI 1640, 10% FBS (Sigma). Each cell construct type was injected into three female NSG mice (NOD.Cg-Prkdcscid Il2rg tm1Wjl/SzJ) 12–14 weeks old from an in-house colony and five female NSGS (NOD.Cg-Prkdcscid Il2rgtm1Wjl Tg(CMV-IL3,CSF2,KITLG)1Eav/MloySzJ) expressing human IL-3, GM-CSF (CSF2) and SCF (KITLG), 8 weeks old, Strain #:013062 from the Jackson Lab, MA. USA. Three days after cell injection doxycycline (hyclate ≥98% (HPLC), Sigma) was administered in the drinking water, 2 mg/mL in 2% sucrose (Sigma) for 3 days followed by 0.2 mg/mL in 2% sucrose replaced three times per week.

Mice were checked daily, weighed and examined at least once weekly to ensure good health. Previous pilot studies indicated that the first cell engraftment health signs are a weak tail that could not be lifted and curled or weak hind legs occurring at approximately 20 days post cell injection. As mice quickly deteriorate following these observations, mice were weighed and examined daily from day 15 after cell transplant by a researcher blinded to the cell type. Mice were humanely killed when either weak tail or hind legs were identified.

### Method details

#### Inhibitor experiments

FLT3 inhibitor Gilteritinib (Selleckchem), CBFβ-RUNX inhibitor (AI-14-91^20^) and RAS inhibitors Abd7 and Ch-3 synthesised by Terry Rabbitts’ group^23^ were dissolved in DMSO to stock concentrations of 1 mM (Gilteritinib) or 50 mM. Inhibitors were prepared at a 10× working concentration in the desired tissue culture media before being added to the target cells after which cells were cultured for the desired period of time prior to endpoint analysis.

#### siRNA transfections

Primary cells were cultured as described above for 7 days prior to the experiment. Cells were collected and concentrated in 300 μL alpha-MEM (Lonza) to 10 × 10^6^ cells/mL. 400 nM of siRNA targeting RUNX1 (Dhamacon J-003926-07, sequence: CGAUAGGUCUCACGCAACA) or mismatch control (sequence: CCUCGAAUUCGUUCUGAGAAG) we added to cells and incubated in an electroporation cuvette (Geneflow) for 5 minutes prior to electroporation at 350V for 10 ms using a BioRad GenePulser Xcell. Cells were counted after 24 h and colony assays prepared. Knockdown of RUNX1 protein was confirmed by western blot as described below.

#### Lentiviral constructs and transduction

Lentiviral vector TCLV2-MEMB-FLAG-VHY6-l10-GFP (TCLV2-RASiDAb),^23^ pCW57.1-dnFOS-GFP^59^ along with an empty vector controls for each vector which contained a GFP insert, were expanded in NEB Stable Competent E Coli (New England Biolabs). The plasmids were transfected into 293T HEK cells by calcium phosphate co-precipitation with four plasmids (TAT, REV, GAG/POL and VSV-G) at a mass ratio of 24 μg:1.2 μg:1.2 μg:1.2 μg:2.4 μg per 150 mm diameter plate of cells. Viral supernatant was collected at 24 h and then each 12 h for a total of four collections prior to concentration by ultracentifugation. Concentrated virus was then added to MOLM14 cells at a 1:1 volume ratio with 8 μg/mL polybrene (Merck) and cells were spinoculated at 1500 xG for 45 min. After 12–16 h media was exchanged.

Transduced cells were sorted by FACS using a FACS Aria (BD Biosciences) to select single cell clones which were then expanded and screened for expression of the RASiDAb or dnFOS RNA by qPCR, the expression of GFP by FACS. RASiDAb clones were also screened by Western Blot to identify clones which decreased phosphorylation of ERK. These clones were chosen for downstream analysis.

#### CellTiter-Glo assay

For viability experiments cells were cultured with inhibitors or with 1.5 μg/mL doxycycline for 3 day (cell lines) or 6 days (primary cell cultures) in opaque white 96 well plates (Grenier). For primary cell co-cultures wells were previously seeded with hMSCs 24 h before addition of primary AML cells. CellTiter-Glo 2.0 was then used to assay cell viability using the manufacturer’s instructions and luminescence was measured using a Centro LB 960 plate reader (Berthold Technologies).

#### Apoptosis assay

Cell lines were cultured for 48 h with inhibitors or 0.1% DMSO as a control, prior to staining for Annexin V and 7-AAD using a FITC Annexin V Apoptosis detection kit with 7-AAD (Biolegend) according to manufacturers instructions. Samples were analysed using a Cytoflex S (Beckman Coulter) and FlowJo software.

#### Trypan Blue assay

Cell lines were seeded at a concentration of 0.2 × 10^6^ cells ml^−1^ with inhibitors or 0.1% DMSO as a control. After 48 h of culture, 10 μL of culture was diluted 1:1 with Trypan Blue (Sigma-Aldrich) and cells were counted using a Cellometer Auto T4 (Nexcelom Bioscience).

#### Flow cytometry analysis of cell markers

Cell lines were cultured for 48 h with inhibitors or 0.1% DMSO as a control prior to staining with antibodies. Primary cells blasts were stained upon defrost. Cells were incubated with antibodies for 10 minutes at 4°C as per the manufacturers’ instructions. Samples were then washed with 0.5% BSA, 2 mM EDTA PBS twice prior to analysis using a Cytoflex S (Beckman Coulter). Analysis was performed using FlowJo software.

#### Cell cycle analysis

Cell lines were cultured for 48 h with inhibitors or 0.1% DMSO as a control prior to cell cycle analysis. Cells were collected and washed twice with PBS. 250 μL of hypotonic buffer (0.5 mg/mL RNase A, 50 μg/mL 7-AAD, 1 mM Tris, 100 μM EDTA, 0.1% Triton X-100, 1 mg/mL sodium citrate) was added to each pelleted sample and vortexed. The samples were left on ice for at least 15 minutes before analysing on a Cytoflex S. Cell cycle analysis was performed using FlowJo software.

#### Colony formation assays

Primary cells were treated with inhibitors or 1.5 μg/mL doxycycline for 24 h in liquid culture prior to seeding at a density of 5000 cells/mL in Methocult Express (StemCell Technologies) with inhibitors or doxycycline added to the media. For cell line experiments cells were seeded at 1000 cells/mL in methocult H4100 (StemCell Technologies) prepared at a 1:1:3 ratio of methocult:FCS:IMDM. Colonies were counted after 12 days (primary cells) or 8 days (cell lines).

#### RNA extraction

Cells were treated with inhibitors in the presence or absence of IL-3 for 24 h prior to RNA extraction using a RNeasy Micro Plus kit (QIAGEN) where less than 50,000 cells were harvested, and a RNeasy Micro Plus kit (QIAGEN) for larger cell numbers.

##### qRT-PCR

Superscript II Reverse Transcriptase (Thermo) was used to synthesise cDNA from 250 ng RNA according to manufacturer’s instructions. Quantitative PCR was performed using SYBR Green mix (Applied Biosystems) at 2× dilution with primers at 100 nM using a 7900HT system (Applied Biosystems). Analyses were performed in technical triplicate using a standard curve from untreated cell cDNA. Primers used for qPCR analysis are listed in Table S2.

#### Western blot

For Western blot analysis of protein expression, RNA extraction was performed on cells treated for 24 h with inhibitors or doxycycline in culture. The flow through from the first step of the extraction was collected and 1.3 mL Acetone was added and samples were incubated at −20°C for 24 h to precipitate protein. This precipitate was pelleted by centrifugation at >13000 xG for 15 min 4°C and the pellet was resuspended in Urea Buffer (8 M urea, 10% Glycerol, 1% SDS, 5 mM DTT, 1 mM Tris pH6.8, 1% PMSF) and quantified using the Bio-Rad Protein Assay manufacturer’s instructions.

3 μg of protein extracts in were run on a 4–20% gradient pre-cast gel (Bio-Rad) and transferred to nitrocellulose using Turbo transfer packs (Bio-Rad). Membranes were blocked with 10% BSA (phospho-primary antibody) or 10% milk in TBS-T (10 mM Tris-HCl pH 7.5, 75 mM NaCl, 0.1% Tween 20) before being incubated at 4°C overnight in 5% BSA/milk TBS-T with primary antibody (anti phospho-p44/42 MAPK (Erk1/2) (Thr202/Tyr204) (1:1000, 9101 Cell Signaling Technology), anti p44/42 MAPK (1:1000, 9102 Cell Signalling Technology), anti-Phospho-FLT3 antibody (Tyr589/591) (30D4) (1:1000, 3464 Cells Signalling Technology), anti-RUNX1 (1:300, 4334 Cell Signalling Technology)). After washing in TBS-T, membranes were incubated in 5% BSA/milk TBS-T with HRP-conjugated anti-rabbit (Cell Signalling Technologies) for 1 h at room temperature. After a further 3 washes in TBS-T, enhanced chemiluminescent reagent (Amersham) was applied and the blot was visualised using a GelDoc system (Bio-Rad). For loading controls, the membranes were stripped using Restore Stripping Buffer (Thermo Fisher Scientific) and GAPDH (ab8245; Abcam) was applied with anti-mouse secondary and visualised as above.

#### LSC competition assay

FACS was carried out using a FACS Aria (BD). LSCs and blasts were identified and sorted using 7-AAD and lineage cocktail-FITC to select lineage-negative viable cells, followed by CD34-PE-Cy7 positive cells and gating CD38-V450 positive blasts and negative LSCs. Each population were divided into two, and the membranes stained with 1) PKH-26 (Merck) and 2) Claret (Merck). The PKH-26 blasts were combined back with the claret LSCs and vice versa, maintaining the original blast:LSC ratio. These cells were then again divided into two and incubated for 6 days as described above with the addition of 100 ng/mL TPO, G-CSF, IL-6, SCF, VEGF, FGFII, and 20 μM EdU, and with or without 20 μM Ch-3, 100 nM gilteritinib and 100 ng/mL IL-3. After 6 days the cells were stained for EdU with the EdU proliferation kit iFluor 488 (Abcam) and flow cytometry was carried out using a CytoFlex S (Beckman Coulter). Analysis was performed using FlowJo software. Cells were gated for viability using forward/side scatter, then LSCs/Blasts using PKH-26 (PE) vs. Claret (APC) and finally EdU positive/negative (FITC). Gating for PKH-26 and Claret was set using cells which were stained in a known proportion of 70:30 PKH-26:Claret and 30:70 PKH-26:Claret.

#### CyTOF panel design and in-house labeling of purified antibodies

The AML CyTOF panel was designed to include cell markers specific for myeloid blasts and cell signaling markers of interest. For most of the targets, antibodies were acquired in pre-conjugated format from the Standard BioTools catalogue. For other targets we performed in-house custom conjugations using the MaxPar X8 antibody-labelling kit (Standard BioTools) following the manufacturers protocol. In addition to lanthanide metals, Indium-115 (Sigma Aldrich) and Platinum- 198 (Fluidigm) were used to label antibodies.

Briefly, X8 polymer stored at −20°C was thawed, resuspended in L buffer and then loaded with 50 mM of lanthanide metal (or In115) at 37°C for 40 min. Metal loaded polymers were washed twice, firstly with L buffer and 25 min centrifugation, and then with C buffer in a 30 mins centrifugation step. During the polymer wash steps 100 μg of purified antibodies were washed with R buffer using a 50 kDa centrifugal unit. Antibodies were then partially reduced with 4 mM TCEP (Fisher) for 30 min at 37°C. Reduced antibodies were twice washed in C buffer. Partially reduced antibodies were mixed with metal-loaded polymer and incubated at 37°C for 90 mins. Conjugated antibodies were washed and centrifuged four times using W buffer. Purified labelled antibodies were finally eluted from the 50 kDa units by a centrifugation step using 100 μL of W buffer and assessed for protein concentration using a NanoDrop spectrophotometer (ThermoFisher). The antibody preparations were returned to the 50 kDa units for a final buffer exchange step with 100 μL PBS antibody stabilization buffer (Candor). For Pt198 labelling we followed the Maecker lab protocol^60^ where platinum directly labels the reduced antibody without the use of polymer. All antibodies were tested at different titres to ascertain the optimal final dilution as follows (format: metal/marker/volume (μl/test)): 89Y/CD45/1.0, 106 Cd/Barcode/0.75, 110 Cd/Barcode/0.75, 111 Cd/Barcode/0.75, 112 Cd/Barcode/0.75, 113 Cd/Barcode/0.75, 114 Cd/Barcode/0.75, 115In/Barcode/0.75, 116 Cd/Barcode/0.75, 143Nd/cPARP/0.7 148Nd/CD34/0.4, 149Sm/p4E-BP1/0.75, 150Nd/pSTAT5/0.5, 152Sm/pAkt/0.6, 153Eu/pSTAT1/0.5, 156Gd/p38/0.5, 158Gd/pSTAT3/0.5, 159Tb/p-cJun/1, 164Dy/IkBalpha/0.5, 165Ho/CD117/0.75, 166Er/NFkB.p65/0.6, 167Er/CD38/0.5, 171Yb/pERK1/2/0.5, 172Yb/ki67/0.75, 173Yb/p-Jnk1/Jnk2/1, 175Lu/pS6/0.5, 176Yb/pCREB/0.4, 198Pt/Barcode/0.75, 103Rh/DNA/500 μM, 194Pt/LIVE/DEAD. cPARP AKT p38.

#### CyTOF experimental workflow

Prior to CyTOF analysis 1 × 10^6^ cells were treated with inhibitors in the presence or absence of IL-3 for 24 h. Cells were fixed in 1% formaldehyde solution (Thermo Scientific) and resuspended to 20-30×10^6^/mL. Antibody cocktail was prepared in excess and filtered through a 0.1 μm centrifugal filter column (Merck Millipore) to remove antibody aggregates.

Samples were initially barcoded by staining cells with metal labelled CD298/B2M antibodies for 20 min at room temperature (RT). Samples were washed twice with MACS buffer. Resuspended cells were then pooled into a single tube and incubated with Tru-Stain Fc blocking solution (Biolegend) for 10 mins at RT. This was immediately followed by incubation with the surface marker antibody cocktail. Staining was performed at RT for 30 min with gentle agitation every 10 min. During the last 2 min of the 30 min incubation, cells were incubated with Cell ID Cisplatin-194 (Pt194). The Pt194 was then quenched with 3 mL MACS buffer. Cells were centrifuged and resuspended in freshly prepared 1.6% paraformaldehyde (Thermo Fisher) and incubated in the dark for 15 mins at RT. Cells were washed in MACS buffer then pelleted cells held on ice for 15 mins. After a further gentle agitation to ensure cells were well dispersed, 1 mL of cold methanol was added to each tube. Cells were incubated at −20°C overnight. The next day tubes were allowed to reach RT then washed twice with MACS buffer. Cells were incubated with antibodies for intracellular targets for 30 mins at RT. Cells were washed with MACS buffer then stained with 500 μM Rh103 DNA intercalator diluted 1:2000 in 500 μl Fix and Perm buffer (Standard BioTools) at 4°C overnight.

Samples were acquired within 72hr of cell staining. Prior to acquisition, the samples were washed once with MACS buffer and then twice with freshly dispensed milliQ deionized distilled water (ddH2O). Cells were then resuspended in ddH2O containing 1/10 diluted four element (EQ) normalization beads (Standard BioTools) and filtered through a cell strainer cap (Thermo Fisher). Cell densities were corrected to be lower than 1×10^6^ cells/mL. Samples were then acquired on a Helios mass cytometer (Standard BioTools) at flow rate of 30 μL/min using a standardized acquisition template following routine tuning and instrument optimization using the HT Helios injector. To ensure absence of sample carryover to the next sample, tubes with milliQ ddH2O (3 min), then wash (nitric acid) solution (3 min) and again miliQ ddH2O (5 min) were run on the instrument in between each sample.

Raw fcs datafiles were (EQ-)bead-normalized using the processing tool in the Fluidigm CyTOF acquisition software. Normalized fcs datafiles were then exported and uploaded to Cytobank software (Beckman Coulter). Each file was cleaned up by a series of manually set gates to exclude normalization beads, non-cellular debris, doublets and dead cells. The processed data was exported into a new experiment where debarcoding was performed to generate individual sample fcs files for further analysis. Processed datafiles were analysed using manual gating. Mean ion count data for each channel was exported after confirming normal distribution using biaxial plots and visualised using heatmaps in R. FCS files of gated cells were exported and read into FlowCore in R, ion counts were log2 transformed and a pseudocount of 1 added, then a Student’s t test performed.

#### DNaseI-seq

DNaseI-seq was performed on purified CD34^+^ or CD117+ blasts from fresh patient samples.^61^ Live cells were added directly to a solution of DNase I in dilute NP40, digested for 3 min at 22°C, and the reactions then terminated by addition of SDS to 0.5%. DNase I (Worthington Biochemical Corporation) was typically used in the range of 2–6 μg/mL using a final 1.5 × 10^7^ cells/mL. Cell lysates were treated with RNAse A for 1 h at 37°C and DNA was isolated by phenol/chloroform extraction. DNase-Seq samples were generated from a size selection of DNase I-digested DNA fragments comprised within a range of 100–250 bp (not including linkers) and libraries were made using a Kapa Hyper prep kit (Roche) according to manufacturer’s instructions. 12–14 cycles of PCR amplification were used and 200–450 bp fragments were size selected by gel electrophoresis on a 1.5% agarose gel. Libraries were validated by qPCR and quantified using the High Sensitivity DNA kit (Agilent) and Kapa Library Quantification kit (Roche) prior to sequencing on a Nextseq 2000 75 using a NextSeq 500/550 High output kit.

#### ATAC-seq

Cells were treated with inhibitors in the presence or absence of IL-3 for 24 h prior to harvest. Omni ATAC-seq was performed as in Corces et al.^62^ Briefly, cells were washed in ATAC resuspension buffer (RSB) (10 mM Tris-HCl pH7.5, 10 mM NaCl and 3 mM MgCl2) and then lysed for 3 minutes on ice in RSB buffer with 0.1% NP-40, 0.1% Tween-20. Then the cells were washed with 1 mL of ATAC wash buffer consisting of RSB with 0.1% Tween-20. Then the nuclear pellet was resuspended in ATAC transposition buffer consisting of 25 μL TD buffer and a concentration of Tn5 transposase enzyme (Illumina) related to the number of input cells, 16.5 μL PBS, 5 μL water, 0.1% tween-20 and 0.01% digitonin and then incubated on a thermomixer at 37°C for 30 minutes. The transposed DNA was then amplified by PCR amplification up to ¼ of maximum amplification, as assessed by a qPCR side reaction. The library was purified using a QIAquick PCR cleanup kit (QIAGEN) followed by ampure (Beckman Coulter) and analysed on a Next Seq 2000 75 using a NextSeq 500/550 High output kit.

#### ChIP-seq

Cells were treated with 10 nM gilteritinib or 1.5 μg/mL doxycycline to induce the Abd-7 RASiDAb in the presence or absence of 10 ng/mL IL-3 for 24 h prior after which 20 million cells were harvested and chromatin was double crosslinked first with 850 μg/mL di(N-succinimidyl) glutarate, followed by 3 washes in PBS, then by incubation in 1% formaldehyde solution (methanol-free from Pierce, Thermo Scientific). 400 mM of glycine (Merck) was added, and cells were washed twice with PBS (Merck) after which pellets were frozen at −80°C.

Crosslinked cells were resuspended at 1 × 10^7^ cells/mL in Buffer A (10 mM HEPES, 10 mM EDTA, 0.5 mM MEGTA, 0.25% Triton- 100, 1× complete mini protease inhibitor cocktail (PIC) (Merck) pH 8.0) and incubated at 4°C for 10 minutes prior to centrifugation at 500 xG for 10 min. This step was repeated with Buffer B (10 mM HEPES, 200 mM NaCl, 1 mM EDTA, 0.5 mM MEGTA, 0.01% Triton X-100, 1× PIC, pH 8.0) and after centrifugation 2 × 10^6^ cells were resuspended in 300 μL IP buffer I (25 mM Tris-HCl, 150 mM NaCl, 2 mM EDTA, 1% Triton X-100, 0.25% SDS, 1× PIC, pH 8.0) and sonicated using a Diagenode Bioruptor Pico sonicator for 11 cycles (30 sec on 30 sec off) before centrifugation for 10 min at 16,000 xG. The supernatant was then collected and 600 μL IP Buffer II (25 mM Tris-HCl, 150 mM NaCl, 2 mM EDTA, 1% Triton X-100, 7.5% Glycerol, 1× PIC, pH 8.0) was added prior to immunoprecipitation.

For immunoprecipitations 15 μL of Dynabeads-Protein G were washed twice with 500 μL 50 mM citrate phosphate buffer pH 5 and resupended in 15 μL citrate phosphate buffer with 4 μg anti-RUNX1 antibody (ab23980, Abcam) or anti-FOS antibody (MA5-15055, Invitrogen) and 0.5% acetyl-BSA before incubation at 4°C for 2 h. After incubation, dynabeads were washed with 500 μL pH 5 citrate phosphate buffer and resuspended in 15 μL citrate phosphate buffer with 0.5% BSA before 555 μL of sonicated chromatin was added and incubated at 4°C for ∼16 h.

After the incubation the dynabeads are washed sequentially with 500 μL of: Wash buffer 1 (20 mM Tris-HCl, 150 mM NaCl, 2 mM EDTA, 1% Triton X-100, 0.1% SDS, pH 8.0) once, Wash buffer 2 (20 mM Tris-HCl, 500 mM NaCl, 20 mM EDTA, 1% Triton X-100, 0.1% SDS, pH 8) twice, LiCl buffer (10 mM Tris-HCl,250 mM LiCl, 1 mM EDTA, 0.5% NP-40, 0.5% Na-deoxycholate, pH 8.0) once, TE/NaCl wash buffer (10 mM Tris-HCl, 50 mM NaCl, 1 mM EDTA, pH 8.0) twice. After these washes DNA was eluted from the dynabeads using 100 μL elution buffer (100 mM NaHCO_3_, 1% SDS). 200 mM NaCl and 500 μg/mL proteinase K were added to the eluant and the sample was reverse crosslinked at 65°C for >4 h. DNA was then purified by ampure (1.8×).

Libraries for next generation sequencing were prepared using a Kapa Hyper prep kit (Roche) according to manufacturer’s instructions. 16 cycles of PCR amplification were used and 200–450 bp fragments were size selected by gel electrophoresis. Libraries were validated by qPCR and quantified using the High Sensitivity DNA kit (Agilent) and Kapa Library Quantification kit (Roche) prior to sequencing on a Nextseq 2000 75 using a NextSeq 500/550 High output kit.

#### RNA-seq

RNA was quantified by nanodrop and QC’d using an Agilent RNA 6000 Pico Kit (Agilent, bioanalyser). Libraries for next generation sequencing were prepared using the NEBnext Ultra II Directional RNA Library Prep Kit for Illumina (NEB) with the NEBNext rRNA Depletion Kit v2 for low RNA input (<100 ng RNA), or the Total RNA Ribo-zero library preparation kit (with ribosomal RNA depletion) (Illumina) for higher RNA input. Libraries were quantified using the High Sensitivity DNA kit (Agilent) and Kapa Library Quantification kit (Roche) prior to paired end sequencing on a Next Seq 2000 (PE 75) with a NextSeq High 150 v2.5 kit.

#### PCR for FLT3-ITD detection

Presence of FLT3-ITD mutations were confirmed by PCR using genomic.^2^ Taq DNA polymerase (Thermo fisher) was used to amplify a 394 bp region of the wild type FLT3 gene using the primers described in Table S2. PCR products were separated on a 1.5% agarose gel with the presence of FLT3-ITD mutations indicated by a larger amplicon than the wild type.

#### Bioinformatics methods

##### ChIP-seq data analysis

Raw sequencing reads were trimmed to remove low quality sequences and adaptors using Trimmomatic v0.39.^43^ Reads were then aligned to the human genome (version hg38) using Bowtie2 v2.2.5^49^ with the parameter --very-sensitive-local. Potential PCR duplicates were identified and removed from alignments using the MarkDuplicates function in Picard tools v2.26.10 (https://broadinstitute.github.io/picard). Peak calling was performed using MACS v2.2.7.1^50^ using the option -B --trackline -q 0.01. The resulting peaks were filtered to remove sites with less than 10 reads at the peak summit or that were found in the hg38 blacklist.^63^ To create a peak union, peaks were first extended by −/+ 100 base pairs from the peak summit, and then merged into a single peak set using the merge function in bedtools v2.30.0.^51^ Differentially bound ChIP peaks were identified by first retrieving the average tag count for each sample from the bedgraph files produced by MACS2, using the annotatePeaks.pl function in Homer with the options -size 200 -bedGraph. These tag counts were then normalized as tag-count per million in R. A peak was considered to be differentially bound if it had a 2-fold difference in normalized tag count between conditions (FOS ChIP) or unnormalized tag count (RUNX1 ChIP).

To create tag-density plots, peaks were first ranked according to fold-difference. The read density in a 2 kb window centered on the peak summit was retrieved using the annotatePeaks.pl function in Homer v4.9.1^52^ with the options -size 2000 -hist 10 -ghist -bedgraph and plotted as a heatmap in Java TreeView v1.1.6r4.^53^

A *de-novo* motif search was carried out within the sets of differentially bound peaks using the findMotifGenome.pl function in Homer. The locations of enriched motifs were then retrieved using the annotatePeaks.pl function in Homer with the -size 2000 -hist 10 -ghist -m options, and plotted using Java TreeView.

##### ATAC-seq data analysis

Single-end reads from ATAC-Seq experiments were trimmed with Trimmomatic and aligned to the human genome (version hg38) using Bowtie2 with the --very-sensitive-local parameter. Potential PCR duplicates were identified and removed from alignments using the MarkDuplicates function in Picard. Peak calling was then carried out using MACS2 with the options --nomodel --shift −100 --extsize 200 -B --trackline. To create a high-confidence peak set that could be used to accurately compare peaks from different samples, alignments from all samples were combined into a single BAM file using the merge function in Samtools v1.12.^55^ Peak calling was then repeated on this merged dataset. Peak positions from each sample/replicate were mapped to these peak positions using the intersect function in bedtools and were used as the reference peak coordinates in all further downstream analyses. Only peaks that were called in both replicates of each condition, and that were not found in the hg38 blacklist were retained for further analysis. Peaks were then annotated as promoter-proximal if found within 1.5 kb of a transcription start site (TSS) using the annotatePeaks.pl function in Homer. Peaks with a distance greater than 1.5 kb were considered as distal elements.

Read counts within peaks were retrieved from the union of all peaks using featureCounts^45^ v2.0.1, and were normalized using the edgeR^48^ package in R v4.1.2. Differential peak accessibility analysis was carried out with Limma-Voom.^46^ A peak was considered to be differentially accessible if it had at least a 2-fold difference between conditions and an adjusted p value <0.1. A *de-novo* motif search was then carried out in the set of differential peaks using the findMotifGenome.pl function in Homer. Heatmaps showing ATAC-Seq read density and enriched motif positions were created in the same way as ChIP-Seq datasets described above.

##### RNA-seq data analysis

Paired-end reads from RNA sequencing experiments were processed using Trimmomatic. Processed reads were then aligned to the human genome (version hg38) using Hisat2 v2.2.1^44^ with default parameters. Read counts were calculated using featureCounts with the options -s 2 -p and using gene models from ensembl as the reference transcriptome. Counts were normalized using the edgeR package in R, and differential gene expression analysis was carried out using Limma-Voom. A gene was considered to be differentially expressed if it had a fold-change of at least 2 and an adjusted p value <0.1. A Kyoto Encyclopedia of Genes and Genomes (KEGG) pathway analysis was carried out for sets of differentially expressed genes using the ClueGO v2.5.0^47^ plugin in Cytoscape v3.9.1.^54^

Hierarchical clustering of RNA-Seq datasets was carried out using log2-transformed normalized count values from edgeR. The Pearson correlation value was calculated for each pair of samples, which were then clustered using complete linkage clustering of the Euclidean distances in R, and shown as a heatmap.

Gene Set Enrichment Analysis (GSEA) was carried out using the GSEA software v2.2.4 from the BROAD institute^56^ using the GSEA pre-ranked algorithm.

##### DNaseI-seq data analysis

Single-end reads were processed for quality control using Trimmomatic and aligned to the human genome (version hg38) using bowtie2 with the option --very-sensitive-local. PCR duplicates were removed from alignments using the MarkDuplicates function in Picard. Peaks were identified using MACS2 with the parameters --nomodel -B --tracklist. The resulting peaks were filtered to remove peaks with less than 10 reads at the summits, as well as peaks that were found in the hg38 blacklist. A peak union was created by combining peaks from all samples into a single dataset. Peaks were then extended by 200 bp in both directions and then merged using the merge function in bedtools.

Differential peak analysis was carried out by first calculating the average tag count in peak regions using the annotatePeaks.pl function in Homer with the options -size 400 -bedgraph with the bedgraph files produced by MACS2. Tag counts were then normalized as tag count per million in R. A peak was considered to be differentially accessible if it had at least a 3-fold difference between samples before and after FLT3i treatment. Read density plots and *de-novo* motif analysis was carried out as described for the ChIP-seq data above.

DNaseI data from healthy PBSCs from Assi et al.^1^ were downloaded from the Gene Expression Omnibus (GEO) using accession GSE108316. These data were processed in the same way as described above.

##### Gene regulatory network (GRN) construction

GRNs were constructed from DNaseI data using in-house python scripts from Coleman and Keane et al. (2023), (see data and code availability section). To do this, DNaseI sites that were gained or lost after FLT3i treatment in patient ITD15 and ITD17 were annotated to their associated genes, that were inferred using promoter-capture HiC data from Assi et al. (2019).^1^ In cases where a peak could not be annotated using the HiC data, closest gene was used. Motif positions were then retrieved from within these peaks using the annotatePeaks.pl function in Homer and exported as BED files using the -mbed option. A network was then created where a node represents transcription factor (TF) genes and their downstream target genes, and an edge is inferred if a DHS linked to that target gene contains a binding motif sequence for a given transcription factor family. As TFs can form large families that can bind to highly similar or identical sequence motifs,^64^ TF gene nodes were grouped into their respective families and the family member with the highest gene expression value (measured using RNA-Seq data in the same patient) was used as the source node for the entire TF family. The resulting networks were then plotted using Cytoscape.

### Quantification and statistical analysis

Statistical analyses were performed using Graphpad Prism v9 software. For pairwise analysis in densitometry, cell viability assays and mouse spleen weight unpaired two-way Student’s t-tests were used to calculate p values. For dose-response curves non-linear fit vs. normalized response was used to calculate the IC50 of the inhibitor in different conditions, we report the average IC50 of 3 independent experiments ± the standard deviation. A hypergeometric test was performed to calculate the expected overlap in venn diagrams using the VennDiagram package in R. For the *in vivo* experiment survival analysis was performed using a log-rank (Mantel-Cox) test. Further details of the statistical tests used can be found in the relevant figure legends.

## Supplemental information

Document S1. Figures S1–S8 and Tables S1 and S2

Data S1. TF matrices for patients with FLT3i relapse, related to Figures 1, 2, 3, S1, and S2 Matrices of the gene regulatory networks of specific regions of open chromatin in samples from patients ITD15 and ITD17 before and after FLT3i treatment. Rows show target genes and columns show TF families, numbers show the number of sites with motifs from that family associated to target genes. Tabs show ITD15-specific sites before Quizartiunib treatment (1), and after (2), ITD17-specific sites before Gilteritinib treatment (3) and after (4), sites upregulated or maintained vs healthy PBSCs in ITD15 (5), ITD (16), and ITD (17).

Data S2. Gene lists and KEGG pathway analysis from primary cell and cell line experiments, related to Figures 3 and S2 Gene lists from all Venn diagram overlap analyses are shown on the first tab. KEGG pathway analysis shown in figures are shown in additional tabs.

Data S3. MOLM14 & MV4-11 RNA-seq results and KEGG pathway analysis, related to Figures 7 and S6 RNA-seq analysis shows deregulated genes in MOLM14 and MV4-11 experiments. KEGG pathway analysis of deregulated genes is also shown.

Data S4. ITD18 RNA-seq results and KEGG pathway analysis, related to Figures 5 and S8 RNA-seq analysis shows deregulated genes in ITD18 experiments. KEGG pathway analysis of deregulated genes is also shown.
