# Supplementary material for: Pharmacological inhibition of RAS overcomes FLT3 inhibitor resistance in FLT3-ITD+ AML through AP-1 and RUNX1
Source: iScience. 2024 Mar 26;27(4):109576. doi: 10.1016/j.isci.2024.109576 (PMC11024925; doi:10.1016/j.isci.2024.109576)
Supplement: Document S1. Figures S1–S8 and Tables S1 and S2 [file mmc1.pdf]

iScience, Volume *27*

Supplemental information

Pharmacological inhibition of RAS overcomes FLT3 inhibitor resistance in FLT3-ITD+ AML through AP-1 and RUNX1

Daniel J.L. Coleman, Peter Keane, Paulynn S. Chin, Luke Ames, Sophie Kellaway, Helen Blair, Naeem Khan, James Griffin, Elizabeth Holmes, Alexander Maytum, Sandeep Potluri, Lara Strate, Kinga Koscielniak, Manoj Raghavan, John Bushweller, Olaf Heidenreich, Terry Rabbitts, Peter N. Cockerill, and Constanze Bonifer

Supplementary Material

1. **Supplementary Figures and Legends**
2. **Supplementary Tables and legends**

Figure S1

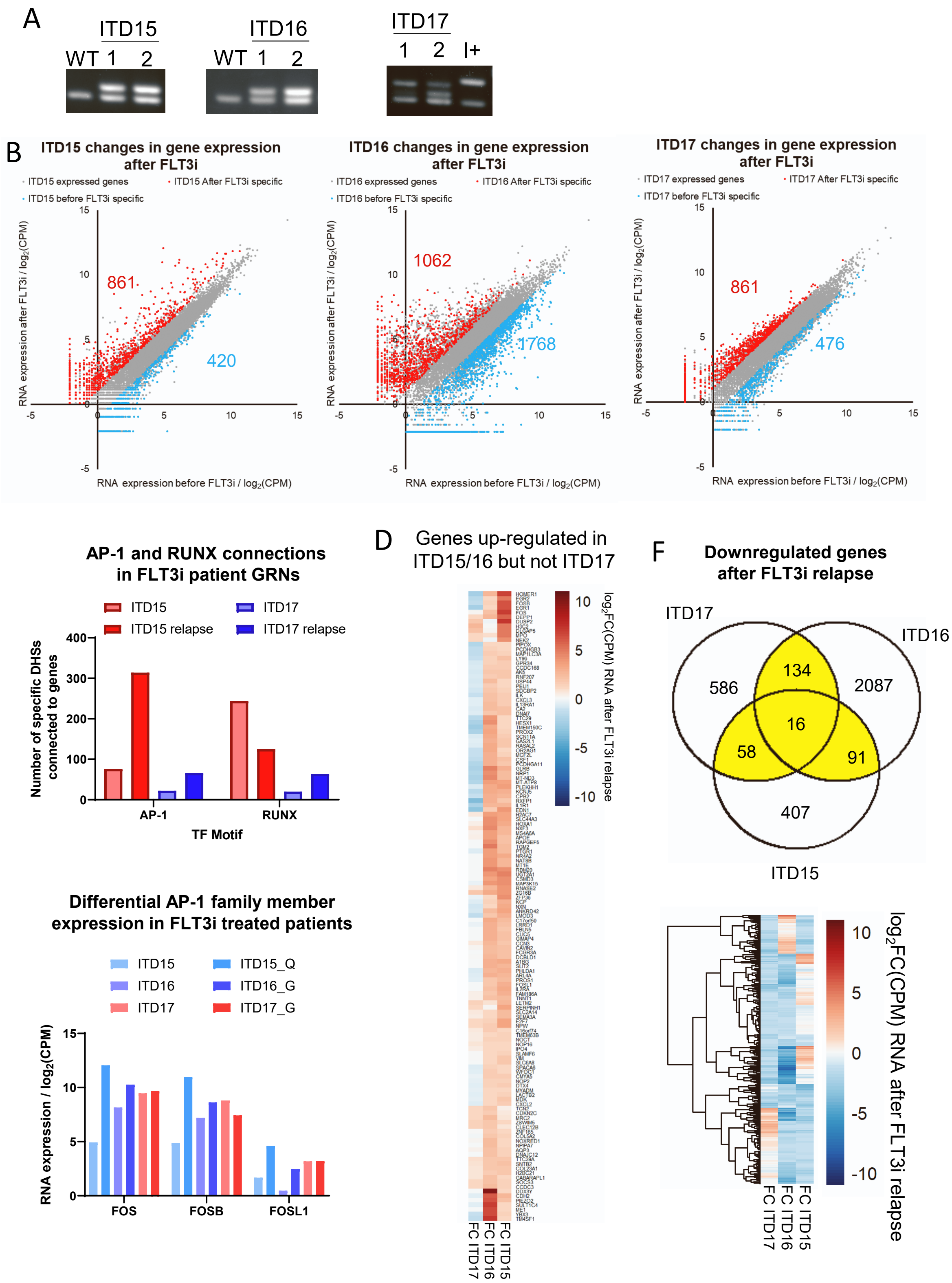

Figure S1: Related to Figure 1. RNA-seq analysis of FLT3 inhibitor treated patients

A: Gels showing results of a PCR assays with FLT3-ITD primers from genomic DNA (gDNA) of patients used in analysis showing that FLT3 ITD is present in samples before (1) and after (2) relapse from FLT3i.

DNA from non-FLT3-ITD Kasumi 1 or gDNA from a FLT3-ITD+ patient sample (I+) were included as controls. B: Scatter plots of RNA expression data (RNA-Seq) of patient samples before and at relapse after FLT3i treatment. Upregulated and downregulated AML specific genes (as compared to healthy PBSCs) are highlighted. C: Histogram showing the number of AP-1 and RUNX1 connections to all genes gained and lost after relapse from FLT3i treatment. D: Heatmap of the expression of deregulated genes highlighted in Figure 1E, showing genes which are upregulated in FLT3i responsive patient but unchanged in the unresponsive patient. The colour indicates fold change in expression of genes after FLT3i relapse. E: Histogram showing expression of FOS family members in FLT3-ITD patient samples before and after relapse from FLT3i treatment as measured by RNA-Seq. F: Venn diagram and unsupervised clustering heatmap of downregulated genes after FLT3i relapse in the indicated samples.

Figure S2

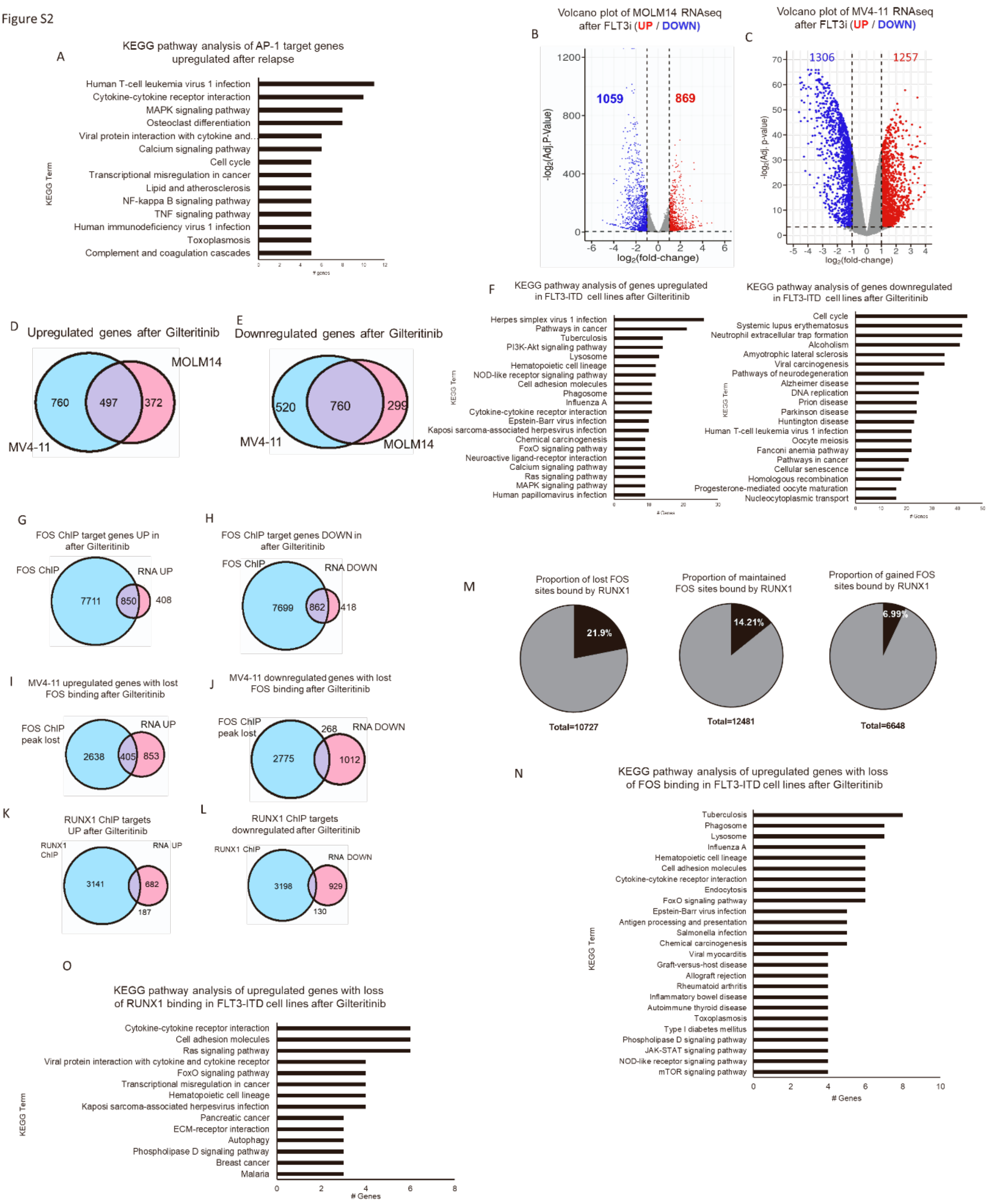

Figure S2: Related to Figure 2. RUNX1 and AP-1 co-operate to deregulate genes after FLT3 inhibition

A: KEGG pathway analysis of AP-1 target genes upregulated after FLT3i relapse in 2 or more patients. B,C: Volcano plots of RNA-seq data from MOLM14 (B) and MV4-11 (C) cells treated with 10 nM Gilteritinib. D,E: Venn diagrams showing overlap of upregulated (D) and downregulated (E) genes in MV4-11 and MOLM14 after FLT3i. F: KEGG pathway analyses of genes upregulated (left) or downregulated (right) in both FLT3-ITD cell lines after Gilteritinib treatment. G,H: Venn diagrams showing the overlap of FOS ChIP target genes and upregulated (G) or downregulated (H) genes in MV4-11 cells after Gilteritinib treatment. I,J: Venn diagrams showing overlap of genes which have lost FOS

binding and upregulated (I) or downregulated (J) genes in MV4-11 cells after Gilteritinib treatment. K&L: Venn diagrams showing overlap of RUNX1 ChIP target genes and upregulated (K) or downregulated (L) genes in MOLM14 cells after Gilteritinib treatment. M: Proportion of FOS peaks in MV4-11 bound by RUNX1 in MOLM14 in lost FOS sites (left), Maintained FOS sites (centre) and gained FOS sites (right) after Gilteritinib treatment. N,O: KEGG pathway analysis of genes upregulated in both FLT3-ITD cell lines after Gilteritinib treatment which are targets of FOS ChIP (N) or RUNX1 ChIP (O).

Figure S3

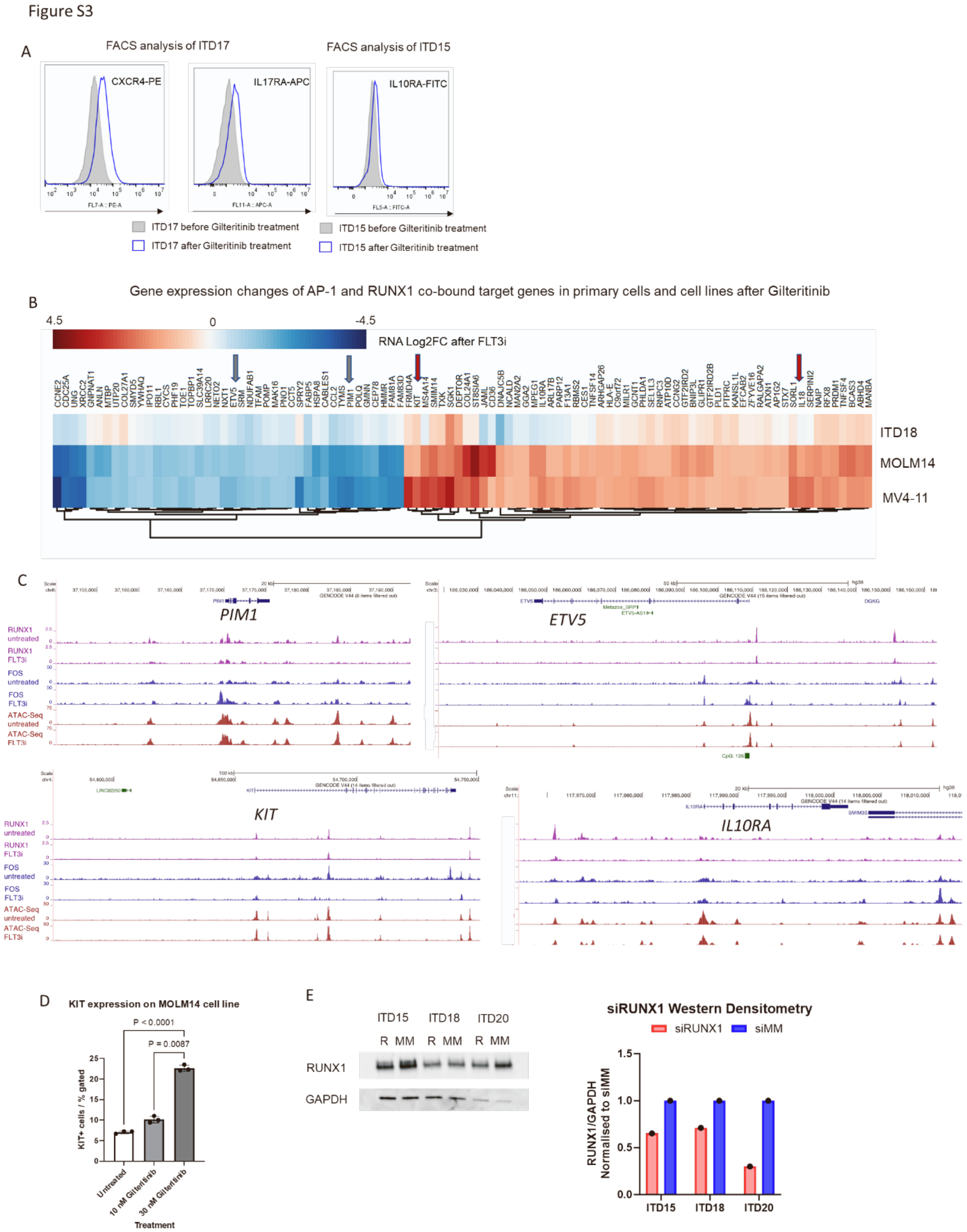

Figure S3: Related to Figure 3. AP-1 and RUNX1 targets are deregulated after FLT3 inhibitor treatment.

A: Histograms from flow cytometry analysis of the indicated cytokine surface receptors on primary cell samples collected before (grey) and after (blue) treatment with FLT3 inhibitors. B: Heatmap of the log2fold change in expression of deregulated genes bound by FOS and RUNX1 in genes with peaks

where FOS was lost after FLT3i. Genes 2-fold deregulated in MV4-11 and MOLM14 and primary cell data from ITD18 after FLT3i are shown. Arrows highlight the genes shown in C. C: USCS browser screen shots of the RUNX1, FOS and ATAC-Seq pattern at the indicated genes. D: Up-regulation of KIT surface expression in MOLM-14 cells after FLT3i treatment measured by flow cytometry. Error bars indicate the ± Standard deviation from (n=3) p values are calculated using Student's t-test. E: Western Blot of protein from samples treated with siRUNX1 for 24 h prior to colony formation assay shown in Figure 3F. Samples were transfected with siRUNX1 (R) or siMM (MM) as a control. The right panel shows the densitometry analysis of RUNX1 signal relative to GAPDH (n=1 for each sample).

Figure S4

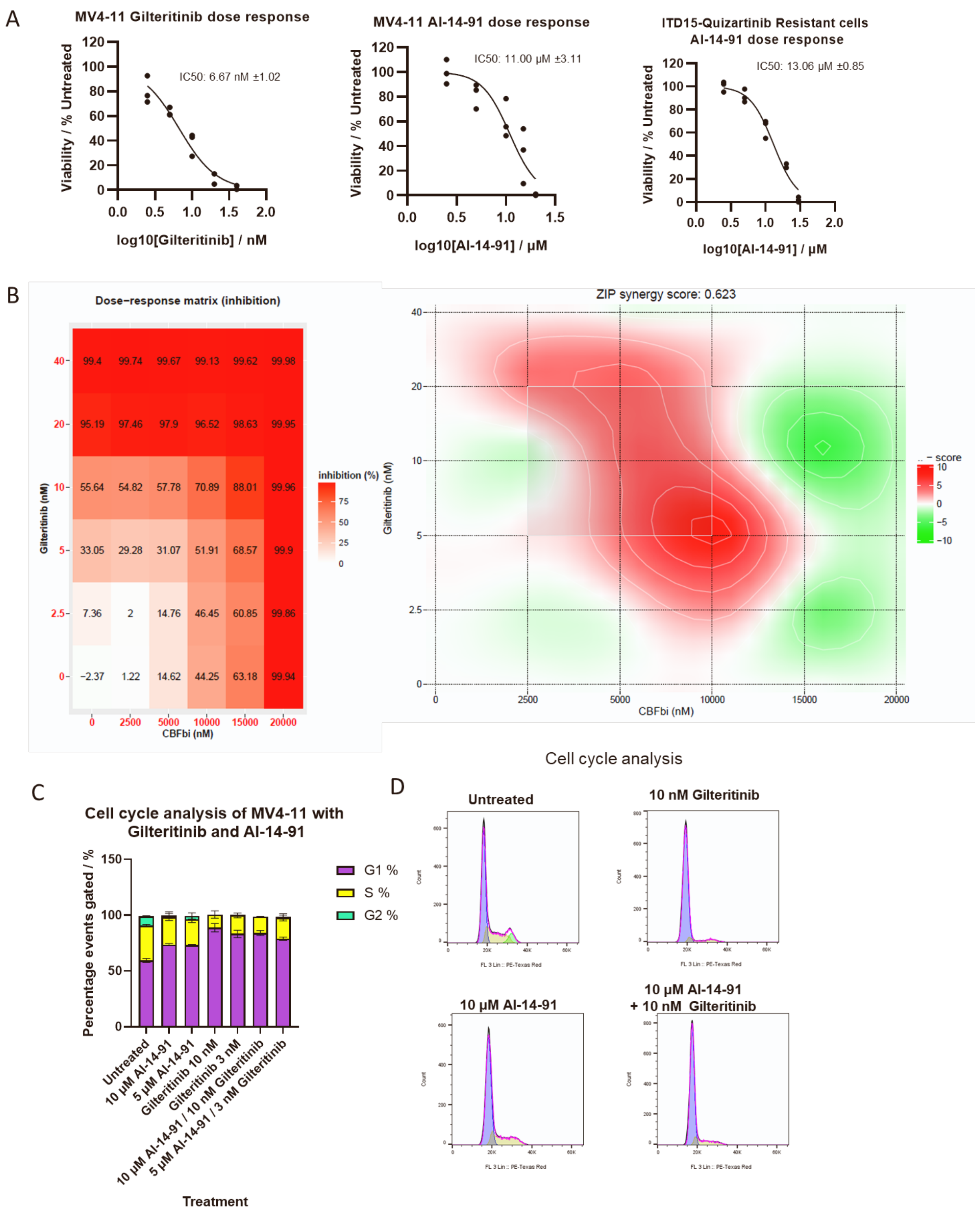

Figure S4: related to Figure 3. RUNX1 is crucial to FLT3-ITD AML survival and shares a pathway with FLT3.

A: Dose response curves of MV4-11 cells treated with Gilteritinib (left) CBFβ inhibitor (right) and ITD15 FLT3i relapse sample primary cells treated with CBFβ inhibitor All IC50 values show the mean IC50 ±

standard deviation (n=3). B: Synergy plot of CBFβi and FLT3i combinations in MV4-11, no synergy is observed, produced using SynergyFinder. C: Histogram showing cell cycle analysis of MV4-11 cells treated with Gilteritinib, CBFβ inhibitor or a combination of the two inhibitors (n=3). D: Examples of cell cycle analysis from (C).

Figure S5

A

Cytokines added to culture	Sample IC50 Gilteritinib / nM		
	ITD15	ITD16	ITD19
No cytokines	24.9 ± 1.65	327.5 ± 179.2	64.2 ± 28.9
20 ng/ml TPO, G-CSF	49.3 ± 16.1	412.9 ± 253.6	52.2 ± 5.9
20 ng/ml TPO, G-CSF, IL-3	98.0 ± 7.21	1108.6 ± 255.9	59.6 ± 5.9
20 ng/ml G-CSF, IL-3, 100 ng/ml TPO	112.8 ± 30.51	962.7 ± 425.1	59.2 ± 7.7
20 ng/ml IL-3, 100 ng/ml TPO, G-CSF	106.2 ± 7.73	1184.8 ± 327.8	58.8 ± 14.9
100 ng/ml TPO, G-CSF, IL-3	140.9 ± 31.82	1056.9 ± 289.0	49.5 ± 2.0
100 ng/ml TPO, G-CSF, IL-3, SCF	95.6 ± 30.38	2006.7 ± 1001.4	59.3 ± 11.9
100 ng/ml TPO, G-CSF, IL-3, SCF, FGFb	121.9 ± 20.7	1308.3 ± 429.3	124.3 ± 29.7
100 ng/ml TPO, G-CSF, IL-3, SCF,FGFb, IL-6	96.7 ± 4.57	1429.7 ± 124.7	123.4 ± 26.5
100 ng/ml TPO, G-CSF, IL-3, SCF,FGFb, IL-6, VEGF	108.4 ± 29.2	1296.7 ± 6.6	162.3 ± 62.7

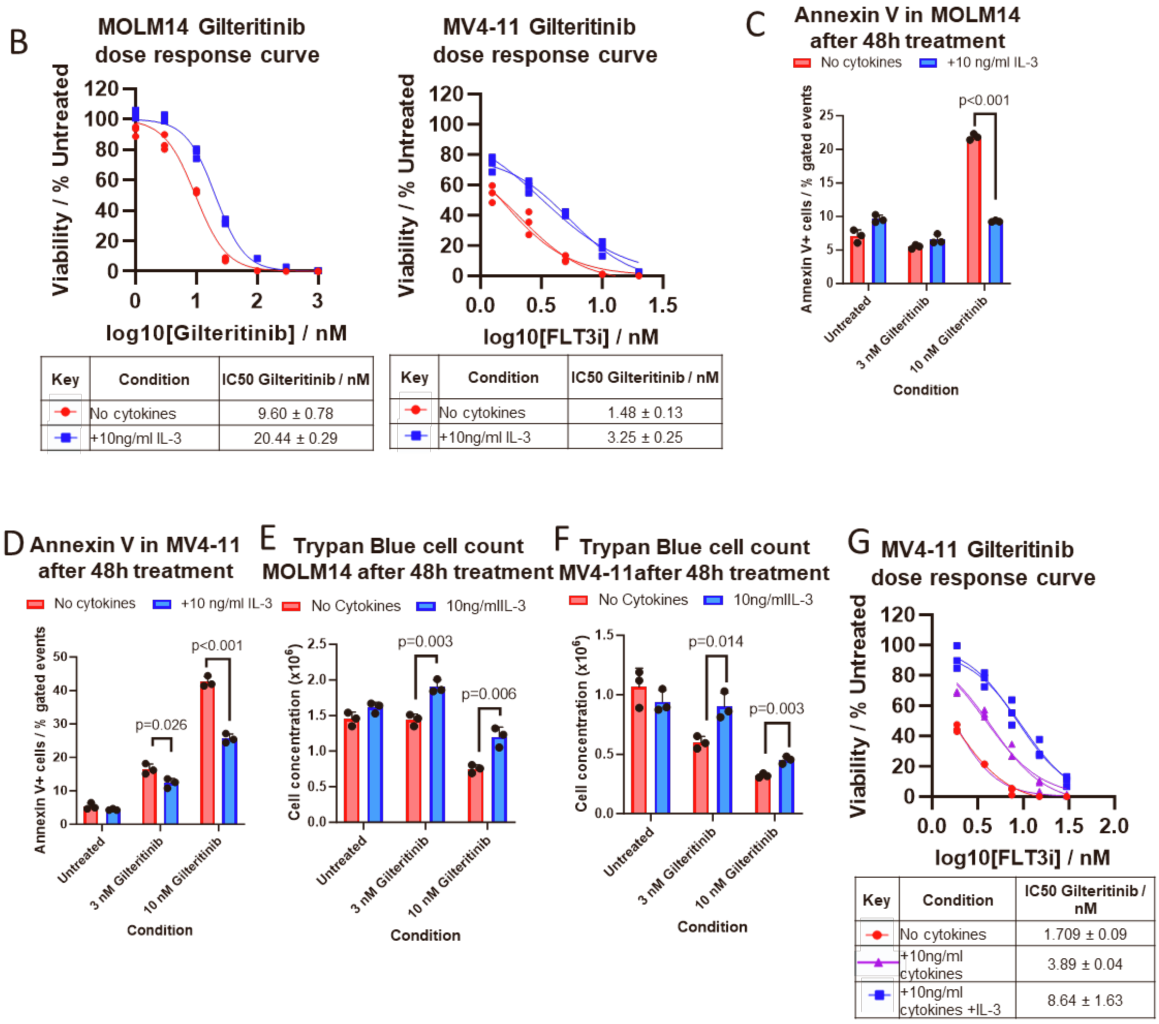

Figure S5: Related to Figure 4. Cytokines induce FLT3 inhibitor resistance in FLT3-ITD+ AML.

A: Table of IC50 from primary cell samples treated with Gilteritinib with cumulative addition of cytokines. Mean IC50 are shown (n=3) +/- standard deviation. The conditions where a log2 increase in

resistance are highlighted in green for each sample. B: Dose response curve depicting the viability of MOLM14 (left) and MV4-11 (right) cells treated with Gilteritinib in the presence and absence of 10 ng/ml IL-3. IC50 values show the mean IC50 ± standard deviation (n=3). C,D: Histogram showing the proportion of Annexin V positive (C) MOLM14 or (D) MV4-11 cells after treatment with Gilteritinib with (blue) or without (red) 10 ng/ml IL-3. E,F: Histogram showing the cell concentration of (E) MOLM14 or (F) MV4-11 cells after treatment with Gilteritinib with (blue) or without (red) 10 ng/ml IL-3. For all histograms (n=3) and significant values are indicated, and were calculated using Student's t-test. G: Dose response curves of MV4-11 treated with gilteritinib in the absence of cytokines (red), with 10 ng/ml TPO, G-CSF, SCF, IL-6, FGFII, IGFII, VEGF (purple) and the same conditions with 10 ng/ml IL-3 (blue). IC50 values show the mean IC50 ± standard deviation (n=3).

Figure S6

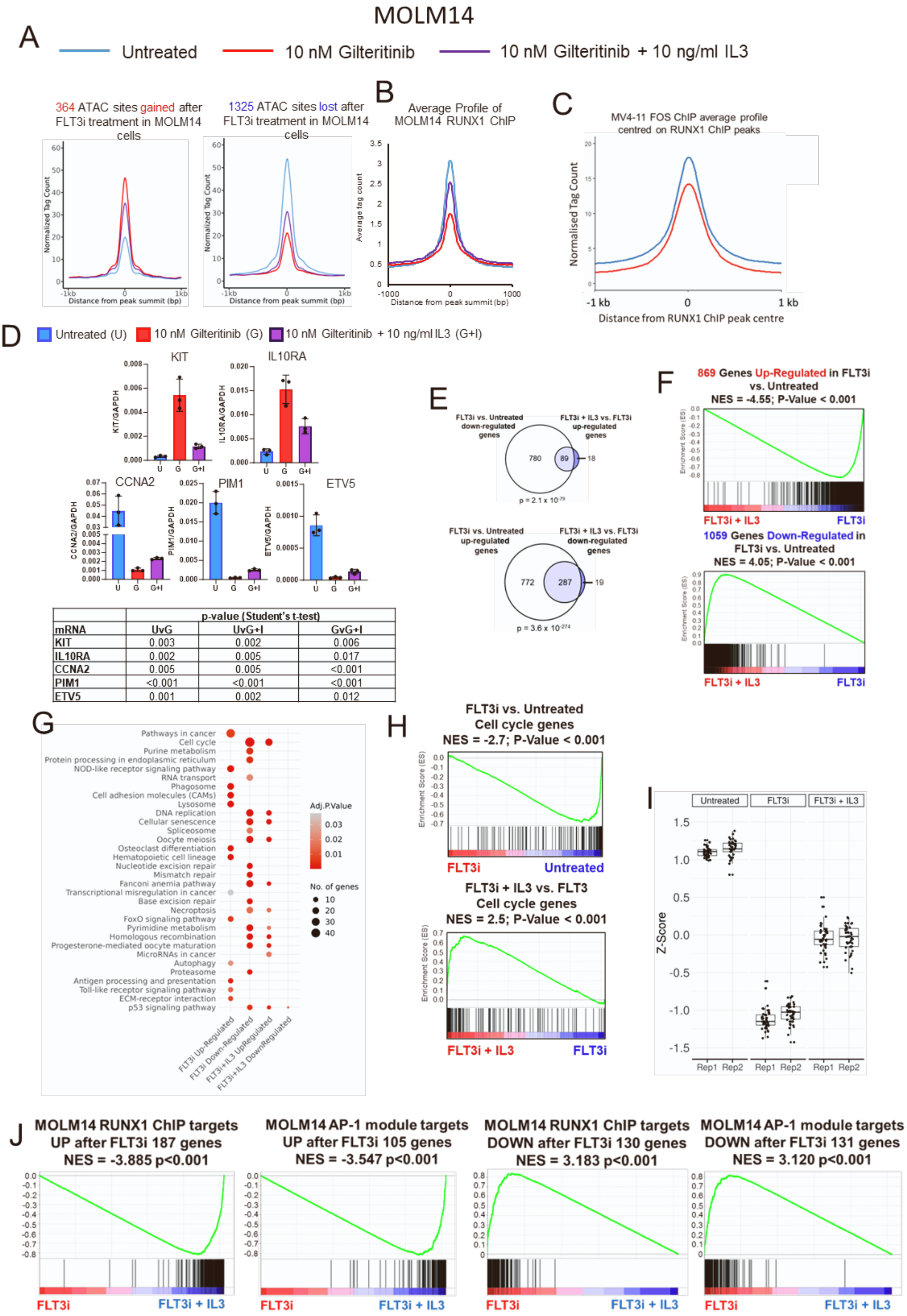

mRNA	p-value (Student's t-test) UvG	UvG+I	GvG+I
KIT	0.003	0.002	0.006
IL10RA	0.002	0.005	0.017
CCNA2	0.005	0.005	<0.001
PIM1	<0.001	<0.001	<0.001
ETV5	0.001	0.002	0.012

Figure S6: Related to Figure 5. IL-3 suppresses genomic and transcriptomic changes which occur after FLT3 inhibiton in FLT3-ITD AML

A: Average profile of ATAC peaks gained and lost in MOLM14 cells after Gilteritinib treatment. B: Average profile of RUNX1 ChIP binding in MOLM14 cells after Gilteritinib treatment. C: Average profile of FOS ChIP binding centred on RUNX1 ChIP peaks in the presence or absence of Gilteritinib. D: Histograms of qPCR analysis of mRNA from MOLM14 cells treated with Gilteritinib +/- 10 ng/ml IL-3. Error bars show standard deviations and p-values calculated using Student's t-test are shown in the table below. E: Venn diagrams showing the overlap of 2-fold upregulated genes after Gilteritinib treatment compared to untreated, and 2 fold downregulated genes in Gilteritinib treated cells with IL-3 (above) and 2 fold downregulated genes after Gilteritinib treatment compared to untreated, and 2-fold upregulated genes in Gilteritinib treated cells with IL-3 (above) in MOLM14 cells. F: Gene set enrichment analysis (GSEA) of genes downregulated (above) or upregulated (below) in Gilteritinib treated MOLM14 cells ranked by the fold change between Gilteritinib treated samples with and without IL-3. G: KEGG pathway analysis of genes deregulated in MOLM14 in Gilteritinib treated cells compared to untreated and Gilteritinib treated cells in the presence and absence of IL-3. H,I: GSEA (H) of cell cycle genes in untreated cells vs Gilteritinib treated cells (above) and Gilteritinib treated cells in the presence and absence of IL-3 (below), with box and whisker plot (I) showing relative expression of cell cycle genes in the three treatments in MOLM14. J: GSEAs in MOLM14 cells treated with Gilteritinib in the presence or absence of IL-3. GSEAs show upregulated genes which are RUNX1 ChIP targets (left) or AP-1 targets (centre left) and downregulated genes which are RUNX1 ChIP targets (centre right) or AP-1 targets (right) in MOLM14 after FLT3 inhibition.

Figure S7

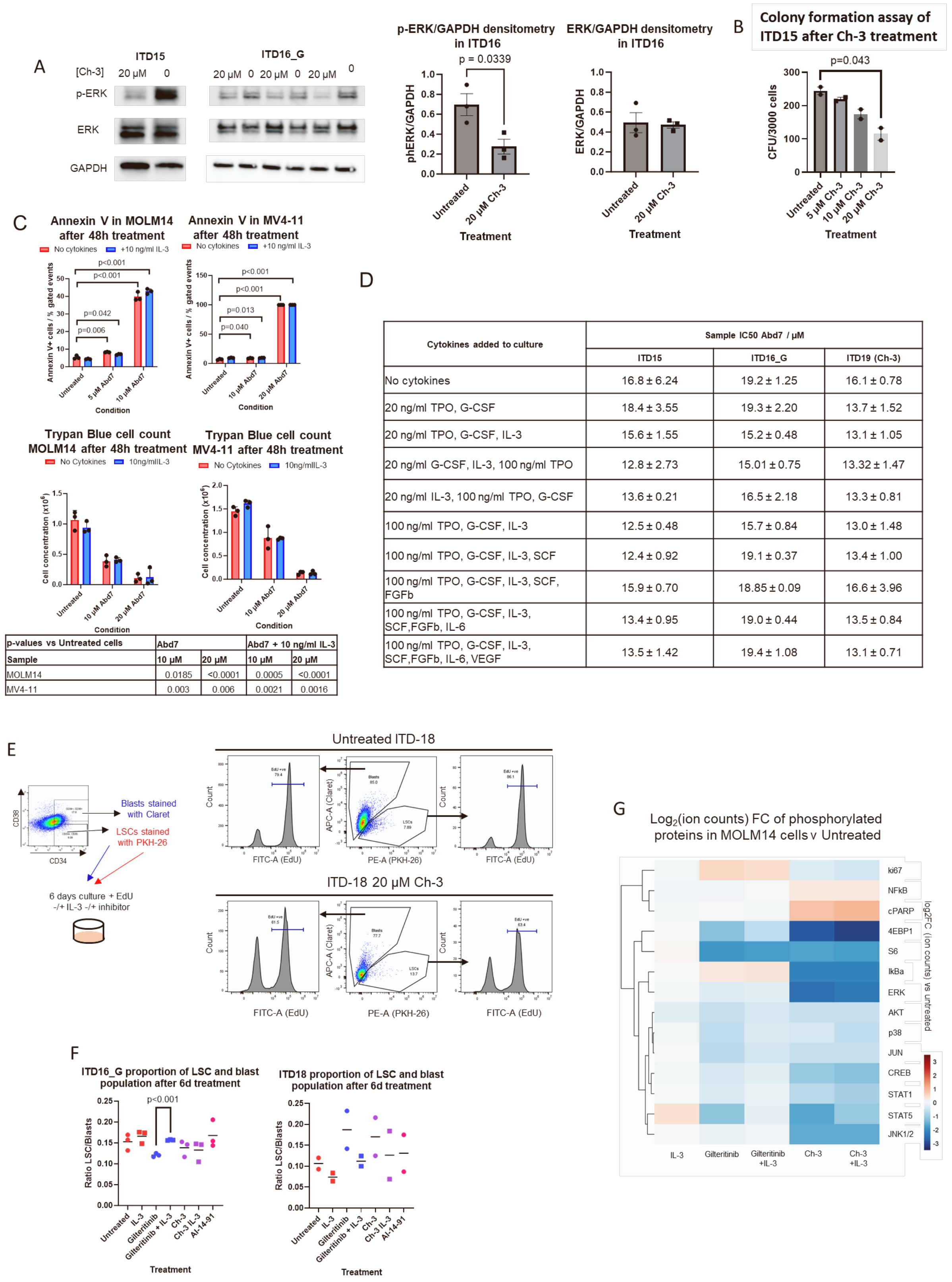

p-values vs Untreated cells	Abd7		Abd7 + 10 ng/ml IL-3	
Sample	10 µM	20 µM	10 µM	20 µM
MOLM14	0.0185	<0.0001	0.0005	<0.0001
MV4-11	0.003	0.006	0.0021	0.0016

Cytokines added to culture	Sample IC50 Abd7 / µM		
	ITD15	ITD16_G	ITD19 (Ch-3)
No cytokines	16.8 ± 6.24	19.2 ± 1.25	16.1 ± 0.78
20 ng/ml TPO, G-CSF	18.4 ± 3.55	19.3 ± 2.20	13.7 ± 1.52
20 ng/ml TPO, G-CSF, IL-3	15.6 ± 1.55	15.2 ± 0.48	13.1 ± 1.05
20 ng/ml G-CSF, IL-3, 100 ng/ml TPO	12.8 ± 2.73	15.01 ± 0.75	13.32 ± 1.47
20 ng/ml IL-3, 100 ng/ml TPO, G-CSF	13.6 ± 0.21	16.5 ± 2.18	13.3 ± 0.81
100 ng/ml TPO, G-CSF, IL-3	12.5 ± 0.48	15.7 ± 0.84	13.0 ± 1.48
100 ng/ml TPO, G-CSF, IL-3, SCF	12.4 ± 0.92	19.1 ± 0.37	13.4 ± 1.00
100 ng/ml TPO, G-CSF, IL-3, SCF, FGFb	15.9 ± 0.70	18.85 ± 0.09	16.6 ± 3.96
100 ng/ml TPO, G-CSF, IL-3, SCF,FGFb, IL-6	13.4 ± 0.95	19.0 ± 0.44	13.5 ± 0.84
100 ng/ml TPO, G-CSF, IL-3, SCF,FGFb, IL-6, VEGF	13.5 ± 1.42	19.4 ± 1.08	13.1 ± 0.71

Figure S7: Related to Figure 6. Pharmacological targeting of the RAS protein family is not vulnerable to cytokine mediated resistance

A: Western blot of ITD15 and ITD16_G cells treated with 20 µM Ch-3 showing phospho-ERK, ERK and GAPDH. Densitometry of p-ERK and ERK are shown in the right hand histograms, (n=3). B: Histogram showing colony formation assay of ITD15 primary cells treated with Ch-3 (n=3). C: Histograms showing (top) the proportion of Annexin V positive, or (bottom) the cell counts of MOLM14 or MV4-11 cells after treatment with Ch-3 with (blue) or without (red) 10 ng/ml IL-3. For all histograms (n=3) and significant values are indicated and were calculated using Student's t-test. D: Table of IC50 from primary cell samples treated with Gilteritinib with cumulative addition of cytokines. Mean IC50 are shown (n=3) +/- standard deviation. E: Representative example of flow cytometry plots from LSC/blast membrane staining experiments. F: Relative proportions of LSC and Blast populations from ITD16_G (n=3) and ITD18 (n=2) LSC membrane labelling experiment. G: Relative signal of phosphorylated signalling proteins detected by CYTOF in MOLM14 cells treated with 10 nM Gilteritinib or 15 µM Ch-3 in the presence or absence of 10 ng/ml IL-3.

Figure S8

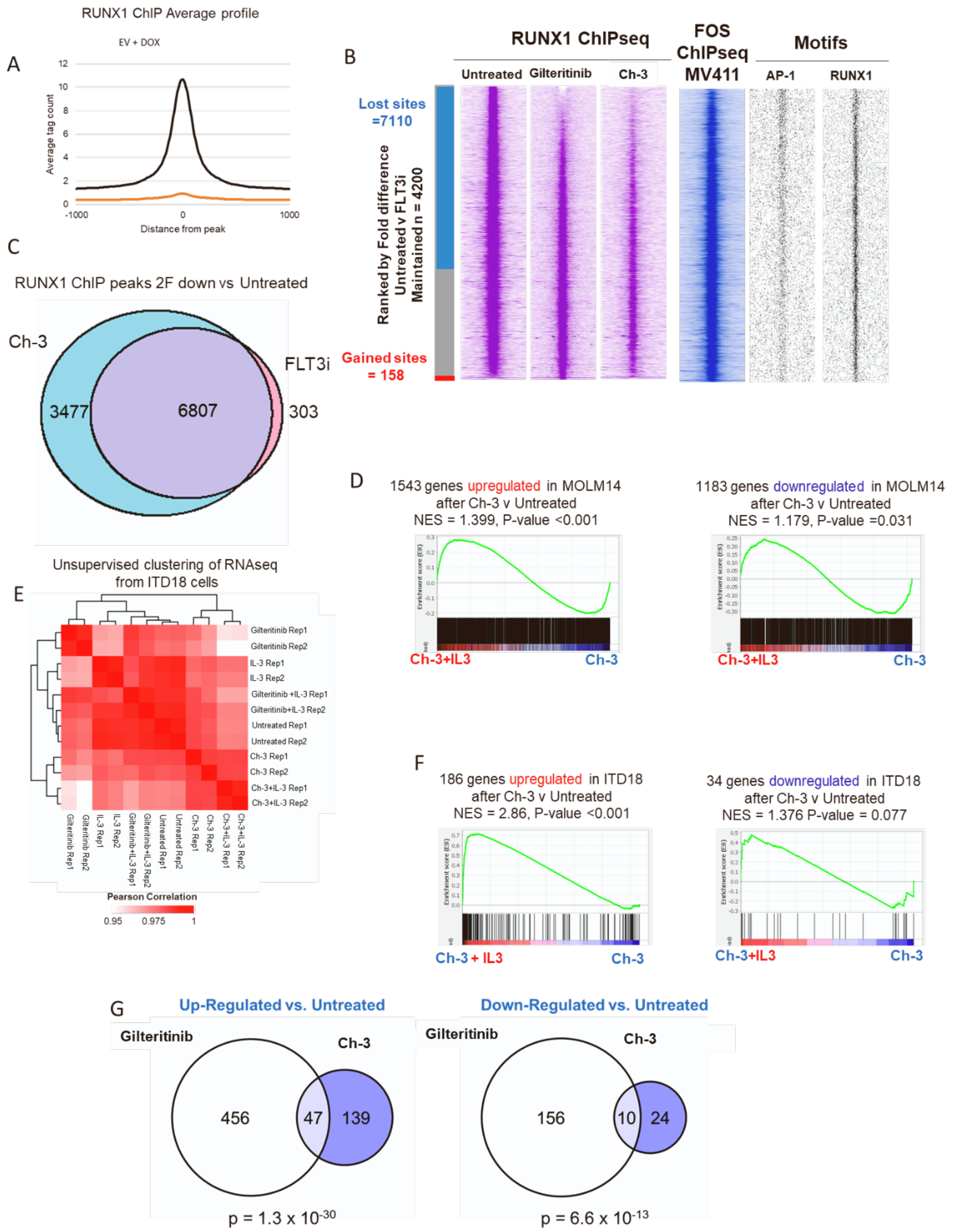

Figure S8: Related to Figure 7. RAS inhibitor mediated gene regulation is not supressed by IL-3

A: Average profile of RUNX1 ChIP in untreated MOLM14 cells and those expressing RAS inhibitor. B: MOLM14 RUNX1 ChIP-peak density plots ranked by fold difference between untreated sample and Gilteritinib treated sample (pink), with MV4-11 FOS ChIP (blue) alongside AP-1 and RUNX1 motif

density (black). C: Venn diagram showing the overlap of RUNX1 ChIP peaks lost in Ch-3 and Gilteritinib treated MOLM14 cells. D: GSEA of deregulated genes in Ch-3 treated MOLM14 primary cells compared to untreated ranked by fold change in ch-3 treated cells in the presence and absence of IL-3 E: Unsupervised clustering of the Pearson correlation values of ITD18 samples treated with Ch-3 or Gilteritinib in the presence or absence of IL-3. F: GSEA of deregulated genes in Ch-3 treated ITD18 primary cells compared to untreated ranked by fold change in ch-3 treated cells in the presence and absence of IL-3. G: Venn diagrams showing overlap of genes 2-fold upregulated (left) or downregulated (right) in ITD18 primary cells with Gilteritinib or Ch-3 treatment.

Supplementary Tables

**Sample**	**Karyotype**	**Detected mutations**	**Sample type**
ITD15	46XY	FLT3-ITD, CHEK2, CUX1,	Relapse
ITD15_rel	46XY	FLT3-ITD, FLT3 D835H, CHEK2, CUX1,	Relapse
ITD16_rel	Complex Karyotype XX	FLT3-ITD	Relapse
ITD17	46XX	FLT3-ITD, NPM1, IDH1	Relapse
ITD17_rel	46XX	FLT3-ITD, NPM1, IDH1	Relapse
ITD18	46XY	FLT3-ITD	Relapse
ITD19	46XY	FLT3-ITD, 2xWT1, CEBPA	Diagnostic
ITD20	46XX	FLT3-ITD NPM1+	Diagnostic

Supplementary Table 1. Related to all figures. Mutations detected in patient samples.
Table of mutations in patient samples detected using the Illumina Trusight myeloid panel.

Target	Forward Primer Sequence	Reverse Primer Sequence
GAPDH	AGGGGCCATCCACAGTCTT	CCTGGCCAAGGTCATCCAT
KIT	ACTGTGGCCGTTATCTGGAA	GAAGTGCCCCTGAAGTACCT
IL10RA	CACAATGGCTTCATCCTCGG	TGGACACAGAACTCTCCCAC
CCNA2	CACTCTACACAGTCACGGGA	AGTGTCTCTGGTGGGTTGAG
PIM1	CATTAGATGGTGCTTGGCCC	GTGTCACTGGTACTCGGGAA
ETV5	AGCTCTGCAGAATCGTGAG	TCTCGATCTGAGGAATGCAG
FLT3 (ITD detection)	GGTGTTTGTCTCCTCTTCATTGT	AAAGCACCTGATCCTAGTACCTT
RUNX1	CCCTCAGCCTCAGAGTCAGAT	AGGCAATGGATCCCAGGTAT

Supplementary Table 2. Related to figures 3, S1, S6. Primer sequences for qRTPCR and FLT3-ITD detection PCR.
Table of sequences for PCR primers
